# Altered visual cortical processing in a mouse model of MECP2 duplication syndrome

**DOI:** 10.1038/s41598-017-06916-3

**Published:** 2017-07-25

**Authors:** Dinghong Zhang, Bin Yu, Jing Liu, Weiqian Jiang, Taorong Xie, Ran Zhang, Dali Tong, Zilong Qiu, Haishan Yao

**Affiliations:** 1 Institute of Neuroscience and State Key Laboratory of Neuroscience, Shanghai Institutes for Biological Sciences, Chinese Academy of Sciences, Shanghai, 200031 China; 2 University of Chinese Academy of Sciences, Shanghai, 200031 China

## Abstract

As an epigenetic modulator of gene expression, Methyl-CpG binding protein 2 (MeCP2) is essential for normal neurological function. Dysfunction of MeCP2 is associated with a variety of neurological disorders. MECP2 gene duplication in human causes neuropsychiatric symptoms such as mental retardation and autism. MeCP2 overexpression in mice results in neurobehavioural disorders, dendritic abnormalities, and synaptic defects. However, how gain of MeCP2 function influences cortical processing of sensory information remains unclear. In this study, we examined visual processing in a mouse model of MECP2 duplication syndrome (MECP2 Tg1 mouse) at 8 and 14 weeks, which were before and after the onset of behavioural symptoms, respectively. *In vivo* extracellular recordings from primary visual cortex (V1) showed that neurons in Tg1 mice at both adult ages preferred higher spatial frequencies (SFs) than those in wild-type (WT) littermate controls, and the semi-saturation contrasts of neurons were lower in Tg1 mice at 8 weeks but not at 14 weeks. Behavioural experiments showed that the performance for visual detection at high SFs and low contrasts was higher in MECP2 Tg1 mice. Thus, MeCP2 gain-of-function in mice leads to higher visual acuity and contrast sensitivity, both at the levels of cortical response and behavioural performance.

## Introduction

Methyl-CpG binding protein 2 (MeCP2), a key epigenetic modulator of gene expression, is widely expressed in the developing and mature brain^1, 2^. The level of MeCP2 is essential for normal neurological function^3^. Mutations in MECP2 gene cause Rett syndrome, a postnatal progressive neurodevelopmental disorder that mostly occurs in girls^4^. Males with duplication of MECP2 display mental retardation, autistic behaviour, and progressive neurological symptoms^5–8^. Similar to human patients, mouse models with MECP2 mutations show progressive neurological dysfunctions recapitulating the symptoms in Rett syndrome^3^; progressive neurological disorders are also found in mice that express MeCP2 at twice the wild-type levels via transgenic insertion of the human MECP2 gene (MECP2 Tg1 mice) or mice that overexpress the mouse MECP2 gene specifically in neurons^9, 10^. Thus, both loss and gain of MeCP2 function lead to abnormalities of the nervous system.

Transgenic mouse models with altered expression of MeCP2 provide powerful tools to investigate the impact of MeCP2 dysfunction on neural circuits. Studies using mice with MeCP2 deficiency have found that pyramidal neurons in slices of the primary somatosensory cortex exhibit reduction in spontaneous firing, decrease in spontaneous excitatory synaptic input and increase in inhibitory drive, and reduction in excitatory synaptic connectivity^11, 12^. It has also been shown that deletion of MeCP2 decreases the excitatory synaptic response in autaptic hippocampal neurons^13^ and decreases the frequency of spontaneous excitatory synaptic transmission in cultured hippocampal neurons^14^. An *in vivo* study has shown that MECP2 knockout adult mice exhibit reduced visual acuity and low neuronal activity in V1^15^. Opposite to the effect of loss of MeCP2 function, enhanced synaptic response is found for cultured hippocampal neurons in mice with MeCP2 overexpression^10, 13^. A recent study in the somatosensory barrel cortex of MECP2 Tg1 mice demonstrates that dendritic arborization, spine density, and spine turnover are abnormal in layer 5 pyramidal neurons^16^. The time course of such dendritic and spine changes approximately coincides with the time course of behavioural symptoms of Tg1 mice^9, 16^. However, little is known about how MeCP2 overexpression affects cortical processing of sensory information.

In this study, we performed *in vivo* electrophysiological recordings to examine the visual response properties of V1 neurons in MECP2 Tg1 mice at two different adult ages, which were before and after the age of symptomatic onset at 10–12 weeks^9^, respectively. At both ages examined, V1 neurons in Tg1 mice exhibited higher spatial frequency (SF) preference compared to those in wild-type (WT) littermates, indicating that MeCP2 overexpression increases the visual acuity of V1 neurons both before and after the onset of behavioural symptoms. The semi-saturation contrast of V1 neurons was lower for Tg1 mice at 8 weeks but not at 14 weeks, indicating that MeCP2 overexpression increases the contrast sensitivity in an age-dependent manner. By training mice to perform visual detection of stimuli that varied in SFs or contrasts, we found that MECP2 Tg1 mice exhibited higher behavioral performance in detecting stimuli at high SFs or at low contrasts. Thus, our results demonstrate that MeCP2 overexpression enhances the sensitivity of visual cortical neurons to high SFs and low contrasts, leading to enhanced behavioural performance in detecting stimuli with high SFs and low contrasts.

## Results

### V1 neurons in MECP2 duplication mice prefer higher SF

**Figure 1:**
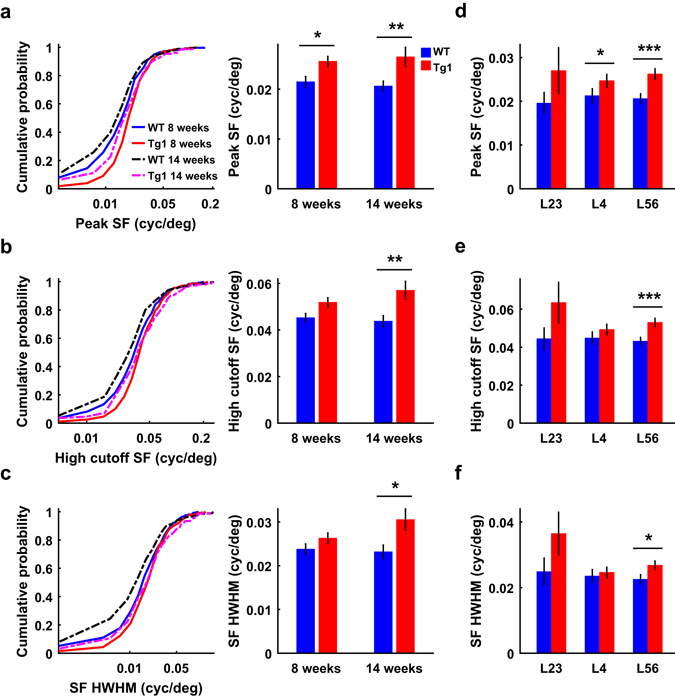
Comparison of spatial frequency preference between V1 neurons in WT and Tg1 mice. (**a**) Cumulative histograms of preferred SFs (left) and mean preferred SFs (right) for V1 neurons in WT and Tg1 mice. (**b**) Cumulative histograms of high cutoff SFs (left) and mean high cutoff SFs (right) for V1 neurons in WT and Tg1 mice. (**c**) Cumulative histograms of HWHMs of SF tunings (left) and mean HWHMs (right) for V1 neurons in WT and Tg1 mice. n = 255 neurons from 27 WT mice at 8 weeks, n = 267 neurons from 22 WT mice at 14 weeks, n = 279 neurons from 29 Tg1 mice at 8 weeks, n = 153 neurons from 13 Tg1 mice at 14 weeks. (**d**) Mean preferred SFs for V1 neurons in each cortical layer for WT and Tg1 mice, combining 8 and 14 weeks. (**e**) Mean high cutoff SFs for V1 neurons in each cortical layer, similar to that described in (**d**). (**f**) Mean HWHMs for V1 neurons in each cortical layer, similar to that described in (**d**). WT mice: n = 12, 77, and 186 for layer 2/3, 4, and 5/6, respectively. Tg1 mice: n = 9, 70, and 247 for layer 2/3, 4, and 5/6, respectively. Only those recordings whose laminar locations could be determined from the CSD analysis were used for laminar analysis. Error bars, ±s.e.m., *P < 0.05, **P < 0.01, ***P < 0.001, two-way ANOVA followed by Tukey’s multiple comparison test for (**a**)–(**c**), Wilcoxon rank sum test with Bonferroni’s correction for multiple comparisons for (**d**)–(**f**).

We used multi-site silicon probes to record the spiking responses of V1 neurons to drifting gratings at different directions and SFs at 100% contrast (Supplementary Fig. S1). For each neuron, we constructed an SF tuning curve using the responses evoked by preferred orientation and fitted the curve with a log Gaussian function (see Methods). V1 neurons in both WT and Tg1 mice exhibited selective responses to different SFs (Supplementary Fig. S1). As shown by Fig. 1a, the cumulative histograms of preferred SFs for V1 neurons were shifted to the right for Tg1 mice compared to those for WT mice at both 8 and 14 weeks. The preferred SFs of V1 neurons were significantly higher in Tg1 mice (8 weeks: 0.026 ± 0.001 cycle/°, s.e.m., n = 279 from 29 mice; 14 weeks: 0.027 ± 0.002 cycle/°, s.e.m., n = 153 from 13 mice) than in WT mice (8 weeks: 0.022 ± 0.001 cycle/°, s.e.m., n = 255 from 27 mice; 14 weeks: 0.021 ± 0.001 cycle/°, s.e.m., n = 267 from 22 mice, P < 0.05, two-way ANOVA followed by Tukey’s test, Fig. 1a). The high cutoff SFs were significantly higher and the half width at half maximal height (HWHM) of SF tuning curves were significantly larger in Tg1 than in WT mice (P = 1 × 10^−4^ and 0.003, respectively, two-way ANOVA, main effect of genotype, Fig. 1b,c). Thus, overexpression of MeCP2 caused a significant shift in V1 neurons’ preference towards higher SF.

To examine the SF preference in different cortical layers, we identified the location of layer 4 by performing current source density (CSD) analysis of the LFP responses evoked by flash stimuli (Supplementary Fig. S2). By combining neurons from mice at both 8 and 14 weeks, we found that the difference in SF preference between WT and Tg1 mice was observed across all cortical layers (Fig. 1d–f). The difference in peak SF was significant in layer 4 and layer 5/6 but not in layer 2/3, likely due to smaller number of cells recorded from layer 2/3 (WT mice: n = 12, 77, and 186 for layer 2/3, 4, and 5/6, respectively; Tg1 mice: n = 9, 70, and 247 for layer 2/3, 4, and 5/6, respectively; P > 0.05 for layer 2/3, P < 0.05 for layer 4 and 5/6; Wilcoxon rank sum test, with Bonferroni correction, Fig. 1d).

We further classified cells as simple or complex based on their responses to drifting gratings at the preferred stimulus^17^. For both simple and complex cells, the preferred SFs and high cutoff SFs were significantly higher in Tg1 mice than in WT mice (P < 0.05, two-way ANOVA, main effect of genotype, Supplementary Fig. S3).

Based on the waveforms of spikes, the neurons were also grouped into broad-spiking and narrow-spiking cells (Supplementary Fig. S4), which correspond to putative excitatory and putative inhibitory neurons, respectively^18^. We found that overexpression of MeCP2 caused significant increase in the peak SF, high cutoff SF, and HWHM of SF tuning for broad-spiking cells (P < 0.05, two-way ANOVA, main effect of genotype) but not for narrow-spiking cells (P > 0.05, two-way ANOVA, main effect of genotype, Supplementary Fig. S5).

By analyzing orientation tunings at the preferred SFs of the neurons, we found that V1 neurons in WT and Tg1 mice did not differ in the distributions of preferred orientations (P > 0.4, *X* ^2^ test) or the HWHMs of orientation tunings (P > 0.3, two-way ANOVA followed by Tukey’s test, Supplementary Fig. S6). Using the global orientation selectivity index (OSI, see Methods) to quantify the orientation selectivity, we found that the OSIs were significantly higher for V1 neurons in Tg1 mice than in WT mice (P = 0.014, two-way ANOVA, main effect of genotype, Supplementary Fig. S6). We further found that there was a weak but significant correlation between the OSI and the preferred SF for V1 neurons in all mice groups except WT mice at 8 weeks (r = 0.27, P = 0.002 for 8-week Tg1 mice; r = 0.12, P = 0.17 for 8-week WT mice; r = 0.42, P = 9.8 × 10^−5^ for 14-week Tg1 mice; r = 0.29, P = 0.004 for 14-week WT mice, Supplementary Fig. S6), consistent with previous reports that orientation tuning is sharper at higher SF^19, 20^ and suggesting that higher OSI for Tg1 mice may be due to higher SF preference of the neurons.

**Figure 2:**
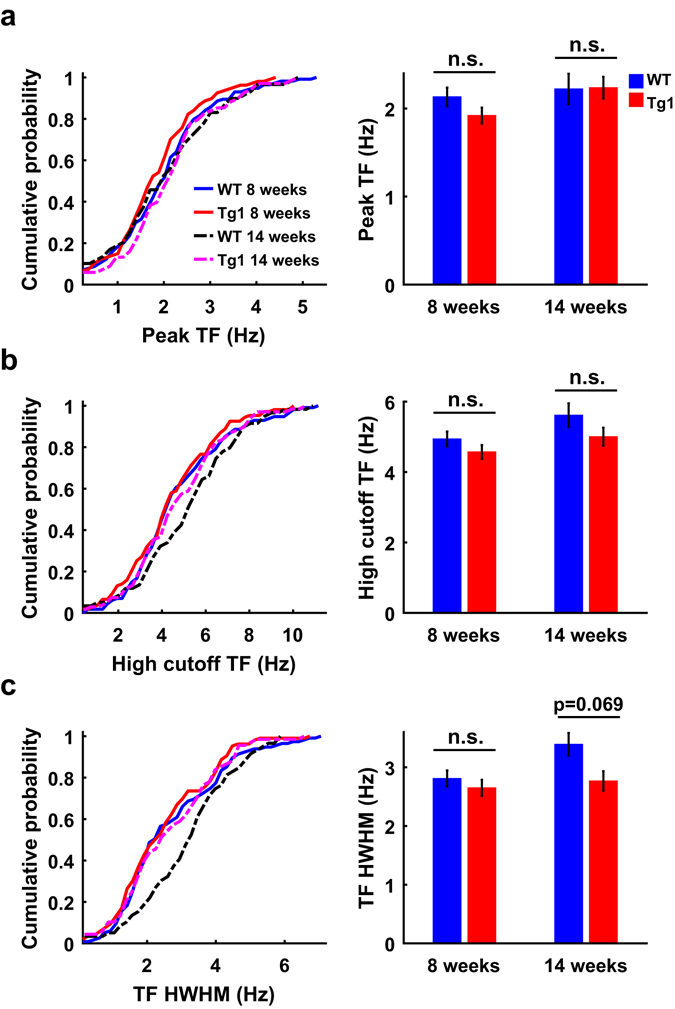
Comparison of temporal frequency preference between V1 neurons in WT and Tg1 mice. (**a**) Cumulative histograms of preferred TFs (left) and mean preferred TFs (right) for V1 neurons in WT and Tg1 mice. (**b**) Cumulative histograms of high cutoff TFs (left) and mean high cutoff TFs (right) for V1 neurons in WT and Tg1 mice. (**c**) Cumulative histograms of HWHMs of TF tunings (left) and mean HWHMs (right) for V1 neurons in WT and Tg1 mice. Error bars, ±s.e.m., P > 0.05, two-way ANOVA followed by Tukey’s multiple comparison test. n = 115 neurons from 16 WT mice at 8 weeks, n = 60 neurons from 11 WT mice at 14 weeks, n = 107 neurons from 17 Tg1 mice at 8 weeks, n = 68 neurons from 17 Tg1 mice at 14 weeks.

Unlike the SF preference, the parameters of temporal frequency (TF) tuning (i.e., Peak TF, high cutoff TF, and HWHM of TF tuning) were not significantly different between V1 neurons in WT and Tg1 mice at either age examined (P > 0.05, two-way ANOVA followed by Tukey’s test, 8 weeks: n = 115 from 16 WT mice, n = 107 from 17 Tg1 mice; 14 weeks: n = 60 from 11 WT mice, n = 68 from 17 Tg1 mice, Fig. 2). Thus, overexpression of MeCP2 did not change the TF preference of V1 neurons.

**Figure 3:**
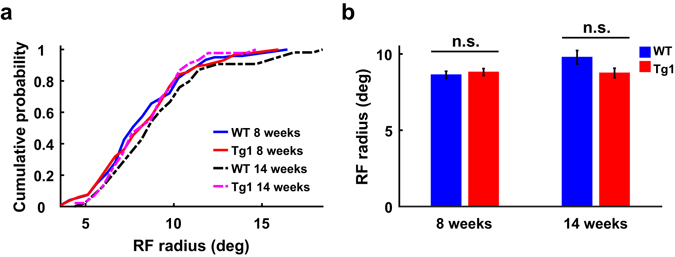
Comparison of RF size between V1 neurons in WT and Tg1 mice. (**a**) Cumulative histograms of RF sizes for V1 neurons in WT and Tg1 mice. (**b**) Mean RF sizes of V1 neurons in WT and Tg1 mice. Error bars, ±s.e.m., P > 0.2, two-way ANOVA followed by Tukey’s multiple comparison test. n = 122 neurons from 16 WT mice at 8 weeks, n = 55 neurons from 11 WT mice at 14 weeks, n = 133 neurons from 16 Tg1 mice at 8 weeks, n = 45 neurons from 12 Tg1 mice at 14 weeks.

To determine whether higher SF preference for V1 neurons in Tg1 mice could be attributed to a reduction in receptive field (RF) size, we measured RF by flashing light squares at different positions in the visual field (Supplementary Fig. S7). We fitted the RF with a two-dimensional elliptical Gaussian, and determined the RF size based on the major and minor axes of the Gaussian fit (see Methods). The RF sizes were not significantly different between WT and Tg1 mice at either 8 or 14 weeks (P > 0.2, two-way ANOVA followed by Tukey’s test, 8 weeks: n = 122 from 16 WT mice, n = 133 from 16 Tg1 mice; 14 weeks: n = 55 from 11 WT mice, n = 45 from 12 Tg1 mice, Fig. 3). Thus, the difference in SF preference between V1 neurons in WT and Tg1 mice could not be attributed to the difference in RF size.

### V1 neurons in MECP2 duplication mice show age-dependent change in firing rate

**Figure 4:**
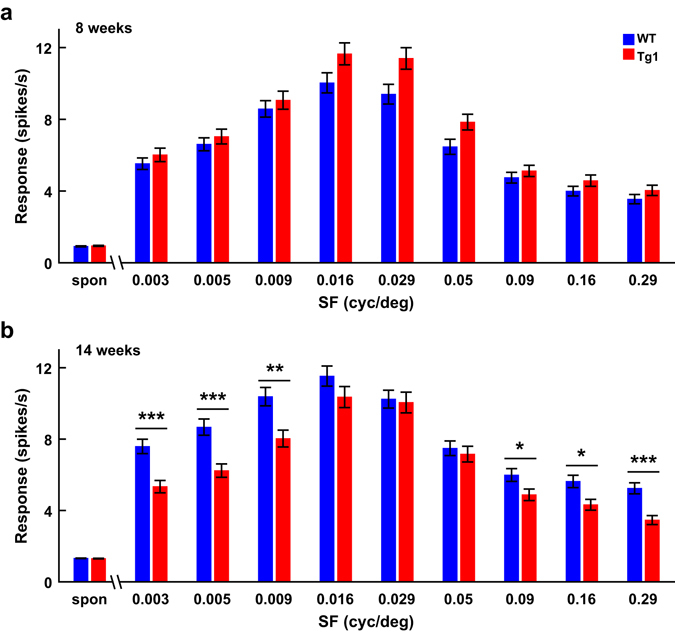
Comparison of spontaneous and evoked responses to each SF between V1 neurons in WT and Tg1 mice. (**a**) Spontaneous and evoked responses for V1 neurons in WT and Tg1 mice at 8 weeks. (**b**) Spontaneous and evoked responses for V1 neurons in WT and Tg1 mice at 14 weeks. Error bars, ±s.e.m., *P < 0.05, **P < 0.01, ***P < 0.001, two-way ANOVA with mixed design, followed by Tukey’s multiple comparison test. n = 255 neurons from 27 WT mice at 8 weeks, n = 267 neurons from 22 WT mice at 14 weeks, n = 279 neurons from 29 Tg1 mice at 8 weeks, n = 153 neurons from 13 Tg1 mice at 14 weeks.

A previous study has shown that both the spontaneous and the evoked responses of V1 neurons are reduced in MECP2 knockout mice compared to WT mice^15^. We found that the spontaneous responses of V1 neurons were not significantly different between WT and Tg1 mice at either 8 or 14 weeks (P > 0.8, two-way ANOVA followed by Tukey’s test, Fig. 4). When we analyzed the neuronal responses to each SF, we found that the evoked responses of V1 neurons in Tg1 mice appeared to be higher at 8 weeks (P = 0.1, two-way ANOVA with mixed design, Fig. 4a) but were significantly lower at 14 weeks (P = 0.02, two-way ANOVA with mixed design, Fig. 4b). Post hoc test after ANOVA revealed that the firing rates to low SFs (0.003–0.009 cycle/°) and high SFs (0.09–0.29 cycle/°) were significantly lower for V1 neurons in 14-week Tg1 mice compared to those in WT mice (P < 0.05, two-way ANOVA with mixed design followed by Tukey’s test, Fig. 4b). Thus, the change in SF preference for V1 neurons in Tg1 mice was associated with an age-dependent change in the evoked responses to a subset of SFs.

### V1 neurons in MECP2 duplication mice show age-dependent change in contrast sensitivity

**Figure 5:**
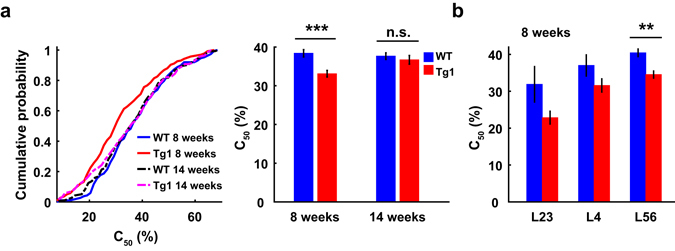
Comparison of contrast sensitivity between V1 neurons in WT and Tg1 mice. (**a**) Left panel, cumulative histograms of C_50_ for V1 neurons in WT and Tg1 mice. Right panel, The C_50_ values of V1 neurons in Tg1 mice were significantly lower than those in WT mice at 8 weeks. The difference in C_50_ between V1 neurons in WT and Tg1 mice was not statistically significant at 14 weeks. n = 187 neurons from 19 WT mice at 8 weeks, n = 258 neurons from 28 WT mice at 14 weeks, n = 259 neurons from 23 Tg1 mice at 8 weeks, n = 173 neurons from 19 Tg1 mice at 14 weeks. (**b**) Mean C_50_ values for V1 neurons in each cortical layer for WT and Tg1 mice at 8 weeks. WT mice: n = 3, 21, and 116 for layer 2/3, 4, and 5/6, respectively. Tg1 mice: n = 6, 48, and 137 for layer 2/3, 4, and 5/6, respectively. Only those recordings whose laminar locations could be determined from the CSD analysis were used for laminar analysis. Error bars, ±s.e.m., **P < 0.01, ***P < 0.001, two-way ANOVA followed by Tukey’s multiple comparison test for (**a**), Wilcoxon rank sum test with Bonferroni’s correction for multiple comparisons for (**b**).

Previous studies have demonstrated that SF could influence the contrast sensitivity of visual cortical neurons^21, 22^. We thus examined whether the difference in SF preference between V1 neurons in WT and Tg1 mice was accompanied by a difference in contrast sensitivity. We measured the responses to drifting gratings at different directions and contrast levels, and obtained the contrast response functions (Supplementary Fig. S8) at the preferred orientations of the neurons. The contrast sensitivity was quantified by C_50_, the contrast at which the response magnitude reached half the maximal response^23, 24^. The C_50_ values of V1 neurons in Tg1 mice were significantly lower than those in WT mice at 8 weeks (WT: 38.4% ± 0.9%, s.e.m., n = 187 from 19 mice; Tg1: 33.1% ± 0.9%, s.e.m., n = 259 from 23 mice, P = 3.9 × 10^−4^, two-way ANOVA followed by Tukey’s test), but the difference in C_50_ between WT and Tg1 mice was not statistically significant at 14 weeks (WT: 37.6% ± 0.9%, s.e.m., n = 258 from 28 mice; Tg1: 36.7% ± 1.1%, s.e.m., n = 173 from 19 mice, P = 0.9, two-way ANOVA followed by Tukey’s test, Fig. 5a). Thus, V1 neurons in Tg1 mice exhibited an age-dependent increase in contrast sensitivity compared to those in WT mice.

The difference in C_50_ between WT and Tg1 mice at 8 weeks was observed across all cortical layers (Fig. 5b), with significant effect in layer 5/6 (P < 0.01 for layer 5/6, P > 0.05 for other layers likely due to smaller number of cells; WT mice: n = 3, 21, and 116 for layer 2/3, 4, and 5/6, respectively. Tg1 mice: n = 6, 48, and 137 for layer 2/3, 4, and 5/6, respectively; Wilcoxon rank sum test, with Bonferroni correction). Significantly lower C_50_ values in Tg1 mice at 8 weeks were observed for both simple and complex cells (P < 0.05, two-way ANOVA followed by Tukey’s test, Supplementary Fig. S9). The difference in C_50_ between WT and Tg1 mice at 8 weeks was significant for broad-spiking cells (P = 3.3 × 10^−5^) but not for narrow-spiking cells (P = 0.96, two-way ANOVA followed by Tukey’s test, Supplementary Fig. S9).

Recent studies have shown that stimulus contrast could influence the orientation selectivity of V1 neurons^25–27^. We thus further analyzed the orientation tunings measured at different contrast levels to examine whether the relationship between orientation selectivity and contrast is affected by MeCP2 overexpression. To quantify the effect of contrast on orientation selectivity, we computed the slope of linear regression between OSI and contrast for each neuron^27^ (Supplementary Fig. S10). For each group of mice, we found that the slopes were significantly larger than zero (P < 1 × 10^−8^, Wilcoxon signed rank test, Supplementary Fig. S10), indicating that mouse V1 neurons exhibited contrast-dependent increase of orientation selectivity, consistent with previous findings^25–27^. The distributions of slopes were not significantly different between V1 neurons in WT and Tg1 mice at either 8 weeks (P > 0.7, Kolmogorov-Smirnov test, n = 95 from 18 WT mice, n = 143 from 22 Tg1 mice) or 14 weeks (P > 0.1, Kolmogorov-Smirnov test, n = 164 from 26 WT mice, n = 111 from 17 Tg1 mice). Thus, overexpression of MeCP2 did not affect the contrast-dependent increase of orientation selectivity in mouse V1.

### V1 neurons in MECP2 duplication mice exhibit higher signal-to-noise ratio for positive-contrast stimuli

**Figure 6:**
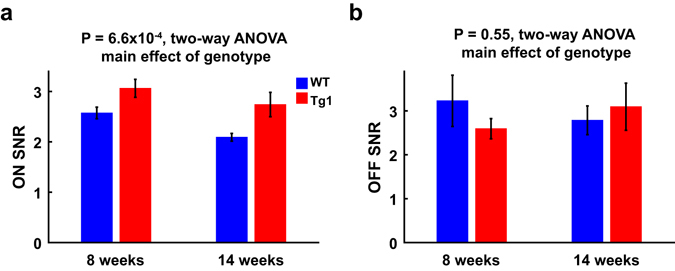
Comparison of ON or OFF RF SNRs between V1 neurons in WT and Tg1 mice. (**a**) Mean SNRs for ON RFs of V1 neurons in WT and Tg1 mice. P = 6.6 × 10^−4^, two-way ANOVA, main effect of genotype. n = 166 neurons from 24 WT mice at 8 weeks, n = 86 neurons from 16 WT mice at 14 weeks, n = 132 neurons from 23 Tg1 mice at 8 weeks, n = 92 neurons from 12 Tg1 mice at 14 weeks. (**b**) Mean SNRs for OFF RFs of V1 neurons in WT and Tg1 mice. P = 0.55, two-way ANOVA, main effect of genotype. n = 60 neurons from 18 WT mice at 8 weeks, n = 59 neurons from 14 WT mice at 14 weeks, n = 69 neurons from 19 Tg1 mice at 8 weeks, n = 33 neurons from 9 Tg1 mice at 14 weeks. Error bars, ±s.e.m.

Previous studies showed that V1 responses to positive-contrast and negative-contrast stimuli were asymmetric, with OFF-dominance in cat and monkey visual cortex^28, 29^ and ON-dominance in mouse visual cortex^30^. We measured the responses of V1 neurons to bright or dark squares on a gray background, and computed signal-to-noise ratios (SNRs) for the ON and OFF RFs, respectively (see Methods). We found that the SNRs for ON RFs were significantly higher in Tg1 mice than in WT mice (P = 6.6 × 10^−4^, two-way ANOVA, main effect of genotype), whereas the SNRs for OFF RFs were not significantly different between the two groups of mice (P = 0.55, two-way ANOVA, main effect of genotype, Fig. 6). Thus, overexpression of MeCP2 caused an increase in the SNR for responses to positive-contrast stimuli.

### SF preference and contrast sensitivity of V1 neurons in F1 hybrid MECP2 duplication mice

**Figure 7:**
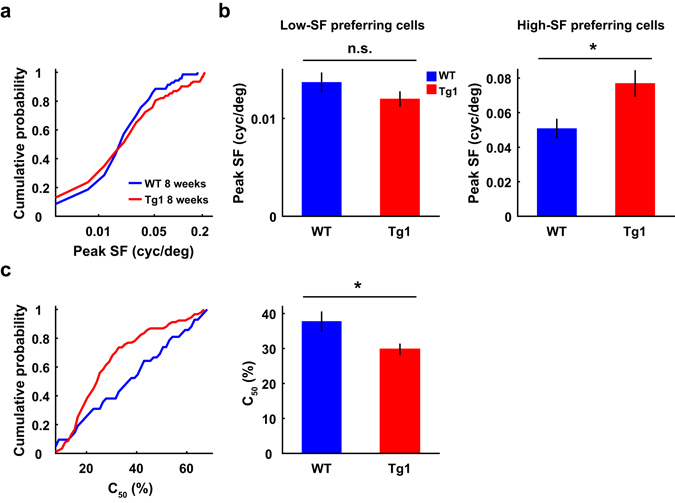
Peak spatial frequency and contrast sensitivity for V1 neurons in mice on a hybrid genetic background. (**a**) Cumulative histograms of peak SFs for V1 neurons in WT and Tg1 mice at 8 weeks. WT: n = 70 neurons from 5 mice; Tg1: n = 124 neurons from 7 mice. (**b**) Left, mean peak SFs for low-SF preferring neurons (n = 34 and 60 for WT and Tg1 mice, respectively). Right, mean peak SFs for high-SF preferring neurons (n = 36 and 64 for WT and Tg1 mice, respectively). (**c**) Left, cumulative histograms of C_50_ for V1 neurons in WT and Tg1 mice. Right, the C_50_ values of V1 neurons in Tg1 mice were significantly lower than those in WT mice at 8 weeks. WT: n = 42 neurons from 5 mice; Tg1: n = 92 neurons from 7 mice. Error bars, ±s.e.m., *P < 0.05, Wilcoxon rank sum test.

The above experiments were performed in mice on an FVB background, which were used in a previous study demonstrating that overexpression of MeCP2 causes progressive neurological symptoms^9^. Mice with different genetic backgrounds differ in visual detection ability and visual acuity^31^, and mice on an FVB background are prone to developing retinal degeneration^32^ (but we have excluded mice with retinal degeneration mutation, see Methods). We thus also examined the responses of V1 neurons in 8-week F1 hybrid mice, which were generated by mating male MECP2 Tg1 mice on an FVB background to female C57BL/6 mice^33^. Unlike the shift in preferred SFs toward higher SF for V1 neurons in MECP2 Tg1 mice on an FVB background, the distributions of preferred SFs were not significantly different between V1 neurons in Tg1 and WT mice on a hybrid background (P = 0.66, n = 70 from 5 WT mice, n = 124 from 7 Tg1 mice, Kolmogorov-Smirnov test, Fig. 7a). Nevertheless, when we separated cells into low-SF preferring and high-SF preferring according to the median of the peak SFs of all neurons, we found that although the peak SFs of low-SF preferring neurons were not significantly different betweenTg1 and WT mice (P = 0.22, n = 34 and 60 for WT and Tg1 mice, respectively, Wilcoxon rank sum test), those of high-SF preferring neurons were significantly higher in Tg1 than in WT mice (P = 0.036, n = 36 and 64 for WT and Tg1 mice, respectively, Wilcoxon rank sum test, Fig. 7b). We also measured contrast response functions for V1 neurons in 8-week mice on a hybrid genetic background. The C_50_ values for V1 neurons were significantly lower in Tg1 mice (WT: 37.7% ± 2.9%, s.e.m., n = 42 from 5 mice; Tg1: 29.8% ± 1.6%, s.e.m., n = 92 from 7 mice, P = 0.02, Wilcoxon rank sum test, Fig. 7c), similar to that observed in mice on an FVB background (Fig. 5). Thus, for 8-week mice on a hybrid genetic background, overexpression of MeCP2 caused an increase in the contrast sensitivity of V1 neurons as well as an increase in the visual acuity for those neurons that preferred high SF.

### MECP2 duplication mice exhibit higher behavioural performance in SF detection and contrast detection tasks

**Figure 8:**
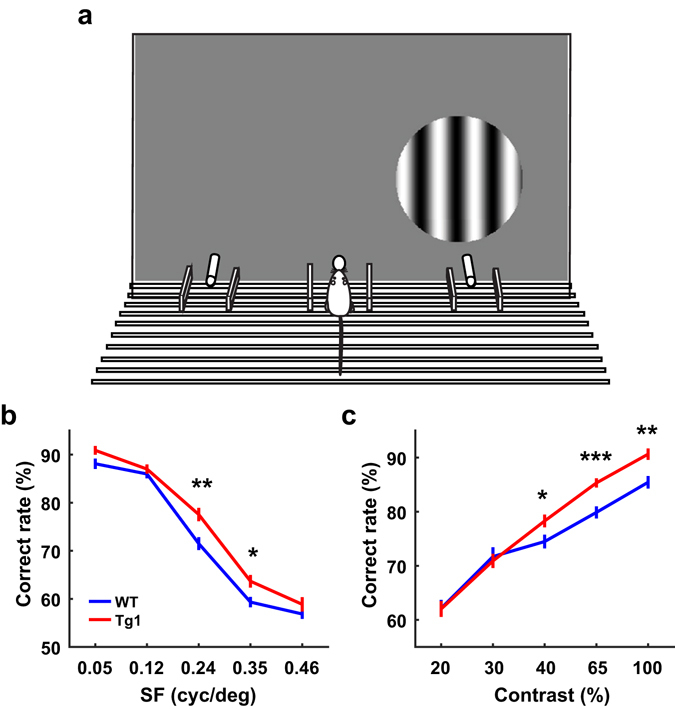
Comparison of behavioural performance between WT and Tg1 mice. (**a**) Schematic illustration of the behavioural apparatus. (**b**) Behavioral performance in visual detection of stimuli that varied in SF (WT mice: n = 8, Tg1 mice: n = 4; P = 0.009, two-way ANOVA, main effect of genotype). (**c**) Behavioral performance in visual detection of stimuli that varied in contrast (WT mice: n = 7, Tg1 mice: n = 6; P = 0.03, two-way ANOVA, main effect of genotype). Error bars, ±s.e.m., *P < 0.05, **P < 0.01, ***P < 0.001. For performance at each SF or each contrast, P value was obtained from two-way ANOVA followed by Tukey’s multiple comparison test.

The above electrophysiological experiments demonstrated that V1 neurons in MECP2 Tg1 mice exhibited enhanced visual acuity at both ages tested and enhanced contrast sensitivity at 8 weeks. To examine the perceptual consequences of the changes in V1 response properties, we trained freely-moving mice to perform a two-alternative forced choice (2AFC) visual detection task^26, 34^. The mouse poked its nose in the center port of a behaviour chamber to trigger the presentation of visual stimulus, which was a static grating on the left or right side of the monitor facing the chamber (Fig. 8a). The mouse indicated its choice by poking its nose into one of the two choice ports. Choosing the port corresponding to the side of the stimulus was considered as a correct choice, which was followed by water reward. Choosing the other port was an incorrect choice, which was followed by a timeout period.

To test the ability of mouse to detect different SFs, we varied the SFs of the grating stimuli (contrast = 100%) from 0.05 to 0.46 cycle/°, similar to those used in a previous behavioural study to examine mouse cortical spatial vision^35^. We found that the performance of visual detection for SFs at 0.24 and 0.35 cycle/° was significantly higher in MECP2 Tg1 mice than in WT mice (P < 0.02, two-way ANOVA followed by Tukey’s test, Fig. 8b). We also tested the ability of mice to detect different contrasts by varying the contrasts of the grating (SF = 0.09 cycle/°) from 20% to 100%. We found that the performance for detecting contrasts ranging from 40% to 100% was significantly higher in MECP2 Tg1 mice than in WT mice (P < 0.05, two-way ANOVA followed by Tukey’s test, Fig. 8c). Thus, MECP2 Tg1 mice exhibited better performance in detecting visual stimuli at high SFs and low contrasts.

## Discussion

In the present study, we compared visual processing between MECP2 Tg1 adult mice that expressed MeCP2 at twice the endogenous level and their WT controls at both 8 and 14 weeks. We found that a prominent effect of MeCP2 overexpression in Tg1 mice was the enhanced visual acuity of V1 neurons. A previous study has demonstrated that MECP2 Tg1 mice exhibit onset of behavioural symptoms around 10–12 weeks of age^9^. We showed that the preferred SF was higher for V1 neurons in Tg1 mice at both 8 and 14 weeks, and the contrast sensitivity was higher for V1 neurons in Tg1 mice at 8 week but not at 14 week. MeCP2 overexpression also caused an enhancement in the SNR of ON RFs and an age-dependent change in the evoked responses to a subset of SFs. Other response properties, including the preferred TF, RF size, distribution of preferred orientation, and the contrast-dependent increase in orientation selectivity, were similar between V1 neurons in WT and Tg1 mice. Consistent with the enhanced visual acuity and contrast sensitivity of V1 neurons, adult MECP2 Tg1 mice showed better behavioral performance in detecting stimuli at high SFs and low contrasts.

Dysfunctions of MeCP2 lead to neurodevelopmental disorders such as Rett syndrome and autism spectrum disorder (ASD)^4–8^. Mouse models of MeCP2 dysfunction exhibit changes in synaptic response and excitatory-inhibitory balance^36, 37^. In mice with MeCP2 deficiency, spontaneous activity is decreased, the balance of excitation and inhibition is shifted to favor inhibition, and excitatory synaptic connectivity is reduced for pyramidal neurons in slices of primary somatosensory cortex^11, 12^. MeCP2 loss disrupts homeostatic synaptic scaling up in rat pyramidal visual cortical neurons^38^ and activity-dependent scaling down in dissociated hippocampal cultures^39^. Loss of MeCP2 function in mice also results in decrease in frequency of spontaneous excitatory synaptic transmission in cultured hippocampal neurons^14^ and reduction in synaptic response of hippocampal glutamatergic neurons^13^. Studies using mouse models of MeCP2 overexpression have found that cultured hippocampal neurons show enhancement in synaptic response and increase in excitatory spontaneous neurotransmission^10, 13^. Although many previous studies have used *in vitro* electrophysiology to examine the mechanisms underlying MeCP2 dysfunction, recent studies are also beginning to use *in vivo* method to examine the effect of MeCP2 dysfunction on developmental plasticity and sensory processing^15, 40, 41^. Nevertheless, there is limited study on how cortical neurons process sensory information in mice with MeCP2 overexpression. Our study may provide insight for understanding the impact of MeCP2 gain of function on cortical computation of sensory information.

A previous study has shown that cortical acuity in V1 is significantly reduced in MECP2 knockout mice, which exhibit a progressive loss of visual acuity after postnatal day P35–40^15^. The reduction of visual acuity in MECP2 knockout mice is preceded by hyperconnectivity of parvalbumin cell circuit and enhanced inhibitory function in V1, and could be rescued by early dark rearing^15^. In our study, we found that the preferred SFs of V1 neurons were significantly higher in MECP2 duplication Tg1 mice at ages both before and after the onset of behavioural symptoms^9^ (Fig. 1). Previous studies in mouse V1 using two-photon calcium imaging or single-unit recording find no spatial clustering of optimal SF^42, 43^. Thus, higher SF preference for V1 neurons in Tg1 mice found in our study is unlikely to be explained by biased sampling of neurons in spatial domains preferring higher SF. In addition to the enhancement in visual acuity, V1 neurons in Tg1 mice also showed increase in orientation selectivity (Supplementary Fig. S6). The enhanced orientation selectivity is likely due to the effect that the orientation selectivity positively correlated with the preferred SF in V1 (Supplementary Fig. S6). Although the spontaneous responses of V1 neurons were similar between Tg1 and WT mice at either 8 or 14 weeks, the evoked responses were significantly affected in Tg1 mice at 14 weeks (Fig. 4), consistent with previous reports that loss or gain of MeCP2 function could cause changes in the balance of excitation and inhibition^36, 37^. Compared to those in WT mice, the evoked responses of V1 neurons in Tg1 mice at 14 weeks were lower for a subset of low and high SFs (Fig. 4), suggesting that the changes in excitation/inhibition balance are both stimulus-dependent and age-dependent. Furthermore, we found that the contrast sensitivity of V1 neurons was higher in Tg1 mice at 8 weeks but similar to that in WT mice at 14 weeks (Fig. 5). A recent study on mice visual perceptual learning reports that enhanced visual acuity and contrast sensitivity in mice are accompanied by an increase in the dendritic spine density in V1^44^. It is interesting to speculate that the age-dependent changes in cortical dendritic structure and spine density observed in Tg1 mice^16, 45^ may contribute to the age-dependent changes in visual responses of V1 neurons. It is also of interest for future study to examine whether the changes of V1 responses in Tg1 mice depend on visual experience.

Since most of the mice used in our study were on an FVB genetic background^9^ that could develop premature retinal degeneration, we have screened for the *Pde6b* ^*rd1*^ mutation affecting vision^32^ so that all mice used in the experiments were not blind. We also recorded from V1 neurons in F1 hybrid mice on FVB and C57BL/6 background (Fig. 7). A consistent effect observed in mice on an FVB and hybrid genetic background was higher contrast sensitivity for V1 neurons in Tg1 mice at 8 weeks (Figs 5 and 7). For mice on a hybrid genetic background, MeCP2 overexpression caused an increase in the visual acuity for subset of V1 neurons that preferred higher SF (Fig. 7).

Patients with MECP2 duplication syndrome display features including anxiety, depression, mental retardation, motor dysfunction, autistic behaviour, and progressive neurological symptoms^5–8^. Transgenic monkeys overexpressing MeCP2 exhibit autism-like behaviours, including repetitive circular locomotion, increased anxiety, and reduced social interactions^46^. Although the major characteristics of ASD are repetitive behaviour and deficits in social interaction and language, previous studies report that abnormalities of sensory perception are also commonly observed in ASD^47, 48^. For example, individuals with ASD exhibit superior performance in tasks requiring local processing for details^49, 50^. Recent studies have found that children with ASD recognize face based on cues of high SF^51^ and autistic patients show biased sensitivity for grating stimuli with high SF^52^. It has been shown that MECP2 Tg1 mice exhibit progressive neurological disorders^9^, some of which are similar to those observed in human patients. Reminiscent of the superior performance in detecting details observed in ASD, MECP2 Tg1 mice also exhibited better performance in detecting visual stimuli at high SFs and low contrasts. By using a mouse model of ASD, our study in MECP2 Tg1 mice suggests that changes in cortical processing of visual information may contribute to the abnormalities of visual perception in subgroups of ASD patients.

## Methods

All procedures complied with the guidelines of the Animal Advisory Committee at the Shanghai Institutes for Biological Sciences, and the protocol was approved by the Animal Care and Use Committee at the Institute of Neuroscience, Chinese Academy of Sciences (protocol number NA-013-2016).

### Animals and surgery

MECP2 duplication Tg1 mice on an FVB background were obtained from the Jackson Laboratory (stock number: 008679)^9^. The mice were maintained by mating duplication males with FVB WT females. Mice were group-housed under a 12:12 h light-dark cycle (light on: 7 am–7 pm). Mice with the retinal degeneration 1 allele of *Pde6b* ^*rd1*^ mutation^32^, which were identified by genotyping PCR (Genotyping protocols database, the Jackson Laboratory), were excluded from the experiments. F1 hybrid mice were generated by mating male MECP2 Tg1 mice on an FVB background to female C57/BL6 mice^33^. Both Tg1 and WT mice used in the experiments were males (20–44 g). Mice on an FVB background, at both 8 and 14 weeks, were used in the anaesthetized physiological experiments; mice on hybrid genetic background at 8 weeks were used in the awake physiological experiments. Mice on an FVB background were used in the behavioural experiments (5–6 weeks of age at the beginning of training).

Details for the number of animals used and the surgery are described in the Supplementary Note.

### Visual stimulation

For electrophysiological recordings, visual stimuli were presented on a 17″ LCD monitor (Dell P170S, mean luminance of 40 cd/m^2^, refresh rate 60 Hz) placed 14 cm away from the animal’s contralateral eye. Gamma correction was used to calibrate the monitor. For behavioural experiments, static sinusoidal gratings (51.5° × 51.5°, assuming that the mouse was at the port facing the stimulus) were presented on a 17″ LCD monitor (Dell E1713S, mean luminance 40 cd/m^2^, refresh rate 60 Hz) placed 14 cm away from the front wall of the behavioural chamber.

The receptive fields of V1 neurons were mapped with sparse noise stimuli, in which a white square (3.6° × 3.6°–5.1° × 5.1°) was flashed on a black background at each of the 16 × 16 positions in a pseudorandom sequence (70 repeats), at an effective frame rate of 30 Hz.

To measure the ON and OFF responses of V1 neurons, a bright or dark square (9.2° × 9.2°) over a gray background was flashed at each of the 8×8 positions in a pseudorandom sequence, with a 200 ms duration and an interstimulus interval of 400 ms^53^. At each location, the bright or dark square was presented 10 times.

Drifting gratings (64° × 64°–71° × 71°) similar to those described in a previous study^18^ were used to measure the tuning properties of V1 neurons. To measure SF and orientation tunings, the stimulus set consisted of drifting gratings (TF = 2 Hz, contrast = 100%) at 12 different directions (spaced at 30°) and 9 different SFs (0.003, 0.005, 0.009, 0.016, 0.029, 0.05, 0.09, 0.16, and 0.29 cycle/°) presented in a random sequence. To measure TF tuning, the stimulus set consisted of 12 different directions and 7 different TFs (SF = 0.025 cycle/°, contrast = 100%, 12 directions spaced at 30°, TFs of 0.25, 0.5, 1, 2, 4, 8, and 16 Hz). To measure contrast response function, we used gratings (TF = 2 Hz, SF = 0.025 cycle/°) at 12 directions (spaced at 30°) and 8 levels of contrasts (5%, 7.7%, 11.8%, 18.1%, 27.7%, 42.5%, 65.2%, and 100%). For all types of grating stimuli used to measure tuning properties, each stimulus was repeated 6 times, and each trial of the stimulus started with a 0.5 s of gray screen, followed by 0.3 s of the first frame of grating and 1.5 or 2 s of the drifting grating. Gray blank condition (mean luminance, ~20 s) was used to estimate the spontaneous firing rate.

To measure the visual responses used for current source density (CSD) analysis, we displayed 200–400 repeats of full-screen flash (100% contrast) for 500 ms with an interval of 500 ms.

For behavioural experiments of visual detection, a static grating was presented on the left or right side of the monitor in each trial. To test the mice’s ability to detect different SFs, SFs of the grating (contrast = 100%) were randomly chosen from 0.05, 0.12, 0.24, 0.35, and 0.46 cycle/° in each trial. To test the mice’s ability to detect different contrasts, contrasts of the grating (SF = 0.09 cycle/°) were randomly chosen from 20%, 30%, 40%, 65%, and 100% in each trial. In each session, the mice performed 350–400 trials, with 35–40 trials for each SF (or contrast) presented on the left or right side. In each trial, the phase of the grating was randomly chosen from 0°, 90°, 180°, and 270°.

### Electrophysiology

Recordings were made with multi-site silicon probes (A1 × 16-3mm-50–177, A1 × 16-5mm-50–177, or A1 × 32-poly2-5mm-50–177, NeuroNexus Technologies) (See Supplementary Note for details).

### Behaviour

Mice were restricted from free access to water 1–2 days before the behavioural training. Details for training are described in the Supplementary Note. In the final step, we measured the mice’s performance in SF detection task (SFs were randomly chosen from 0.05, 0.12, 0.24, 0.35, and 0.46 cycle/°, contrast = 100%) or contrast detection task (contrasts were randomly chosen from 20%, 30%, 40%, 65%, and 100%, SF = 0.09 cycle/°).

### Analysis of neuronal responses

For the responses to 16 × 16 sparse noise stimuli, we binned the spikes at stimulus frame rate and obtained the spatiotemporal RF by computing the spike-triggered stimulus average at a range of time delays^54^. We computed the variance of the spatial RF at each time delay and defined an SNR as the ratio of the maximum variance to the mean variance at 333–500 ms relative to spike occurrence^55^. A cell was included in the analysis of RF size if the SNR was >1.5. The spatial RF map at the delay of peak variance was used for further analysis. We fitted the spatial RF map with a two-dimensional elliptical Gaussian:

1 $$f(x,y)=B+A\exp (-\frac{{[(x-{x}_{0})\cos \theta +{y}_{0}\sin \theta ]}^{2}}{2{\sigma }_{x}^{2}}-\frac{{[-(x-{x}_{0})\sin \theta +(y-{y}_{0})\cos \theta ]}^{2}}{2{\sigma }_{y}^{2}})$$

where *B* is the baseline, *A* is the response amplitude, (*x*_0_, *y*_0_) is the center of RF, *θ* is the orientation of the main elliptical axis, and *σ*_*x*_ and *σ*_*y*_ are the standard deviations of the two axes^56^. The fitting error was computed as^57^:

2 $$E=\frac{\sum {({R}_{measure}-{R}_{fit})}^{2}}{\sum {({R}_{measure}-\bar{R})}^{2}}$$

where *R*_*measure*_ and *R*_*fit*_ are the measured and fitted responses at each position, respectively, and $\bar{R}$ is the measured response averaged over all positions. To quantify RF size, we first used the standard deviations of the major and minor axes to compute the area of the elliptical Gaussian, then treated the area as circular and computed the diameter of the circle^58^. Only those cells for which the fitting error <0.4 were included in the analysis of RF size.

For drifting gratings, spike rate to each stimulus was calculated by averaging the responses during the drifting period over all trials. The spontaneous response was computed as the mean firing rate during the gray screen condition. Details for the analyses to obtain orientation/SF/TF tuning curve and contrast response function are described in the Supplementary Note. We subtracted the spontaneous rate from each tuning curve^18^. We determined whether the neurons were visually responsive by calculating the *t* statistic (mean evoked rate divided by s.e.) for the responses to the optimal stimulus^59^. Only those units with *t* > 2 and peak evoked firing rate >2 Hz were included in the subsequent analyses^59^.

The orientation tuning curve was fitted by the sum of two modified von Mises functions^58, 60^:

3 $$R(\theta )={A}_{0}+{A}_{1}\exp (k[\cos (\theta -{\varphi }_{1})-1])+{A}_{2}\exp (k[\cos (\theta -{\varphi }_{2})-1])$$

where *R*(*θ*) is the response at orientation *θ*, and *A*_0_, *A*_1_, *A*_2_, *k*, *ϕ*_1_, *ϕ*_2_ are free parameters. Preferred orientation and HWHM (i.e., half width at half maximal height) was extracted from the fitted curve^58^.

We computed a global measure of orientation selectivity index (*OSI*)^25, 61^ as:

4 $$OSI=\sqrt{{(\sum _{i}(R({\theta }_{i})\sin (2{\theta }_{i})))}^{2}+{(\sum _{i}(R({\theta }_{i})\cos (2{\theta }_{i})))}^{2}}/\sum _{i}R({\theta }_{i})$$

where *θ*_i_ is the angle of the drifting direction of the grating and *R*(*θ*_i_) is the response at angle *θ*_i_.

The SF tuning was fitted with a log Gaussian function using the following equation^58^:

5 $$R(f)=B+A\exp (\frac{-1}{2{s}^{2}}\,\mathrm{log}\,{(\frac{f+o}{p+o})}^{2})$$

where *f* represents SF, and *B*, *A*, *s*, *o*, *p* are free parameters. From the fitted curve we extracted the peak frequency, the high cutoff SF (the SF at the half-maximal response on the high frequency side of the peak), and HWHM^58^. The TF tuning curve was fitted with the same equation used for SF tuning curve^58^. The contrast response function was fitted with the Naka-Rushton equation^23, 24^:

6 $$R(C)=B+{R}_{{\rm{\max }}}\frac{{C}^{n}}{{C}_{50}^{n}+{C}^{n}}$$

where *C* represents contrast, *R*_max_ represents maximal response, *B* is the baseline, C_50_ is the semi-saturation contrast (the contrast at which half of maximal response is evoked), and *n* is a parameter that defines the steepest slope of the contrast response function.

For the analysis of SF tuning (Figs 1 and 7), TF tuning (Fig. 2), orientation tuning (Supplementary Fig. S6), or contrast response function (Figs 5 and 7), only those cells for which the fitting error <0.4 were included. To analyze the relationship between OSI and preferred SF (Supplementary Fig. S6), we included those neurons in which the fitting error <0.4 for both orientation tuning and SF tuning. For the analysis of the slope of linear regression between OSI and contrast (Supplementary Fig. S10), we included those cells in which the fitting error <0.4 for the orientation tuning at the contrast that produced peak response.

Details for other analyses, including SNR for the ON or OFF RF, CSD analysis, and cell classification (simple and complex cells, broad-spiking and narrow-spiking cells), are described in the Supplementary Note.

### Analysis of behavior

To quantify the performance in the 2AFC task, we calculated the percentage of rightward (or leftward) choices for those trials in which the grating stimulus was presented on the right (or left) side, and averaged the two percentages.

### Statistics

Data are presented as mean ± s.e.m. in the main text and figures. To determine statistical significance, two-way ANOVA followed by Tukey’s multiple comparison test, Wilcoxon signed rank test, Wilcoxon rank sum test (with Bonferroni’s correction for multiple comparisons if necessary), *X* ^2^ test, and Kolmogorov-Smirnov test were used in data analysis. Differences between datasets with P values < 0.05 were considered statistically significant.

### Data Availability

The datasets generated and analysed during the current study are available from the corresponding author on reasonable request.

## Electronic supplementary material

Supplementary Information
