# Supplementary material for: Altered visual cortical processing in a mouse model of MECP2 duplication syndrome
Source: Sci Rep. 2017 Jul 25;7:6468. doi: 10.1038/s41598-017-06916-3 (PMC5526895; doi:10.1038/s41598-017-06916-3)
Supplement: Supplementary file 1 — Supplementary Information [file 41598_2017_6916_MOESM1_ESM.pdf]

Supplementary Information

Altered visual cortical processing in a mouse model of MECP2 duplication syndrome

Dinghong Zhang[1,2], Bin Yu[1,2], Jing Liu[1,2], Weiqian Jiang[1,2], Taorong Xie[1], Ran Zhang[1,2], Dali Tong[1,2], Zilong Qiu[1] & Haishan Yao[1,*]

[1]Institute of Neuroscience and State Key Laboratory of Neuroscience, Shanghai Institutes for Biological Sciences, Chinese Academy of Sciences, Shanghai 200031, China

[2]University of Chinese Academy of Sciences, Shanghai 200031, China

This Supplementary Information contains:

Supplementary Note

Supplementary Figures S1–S10

Supplementary Note

1. Animals and surgery.

For mice on an FVB background, a total of 69 MECP2 Tg1 mice (n = 45 and 24 at 8 and 14 weeks, respectively) and 77 WT littermate controls (n = 42 and 35 at 8 and 14 weeks, respectively) were used in the physiological experiments, and a total of 6 MECP2 Tg1 mice and 8 WT mice were used in the behavioural experiments (aged at 5 – 6 weeks at the beginning of behavioural training). For mice on hybrid genetic background, a total of 7 MECP2 Tg1 mice and 5 WT mice (both at 8 weeks) were used in the physiological experiments. Some mice at 14 weeks exhibited aggressive and hypoactive behaviours similar to those described in previous reports for Tg1 mice[1], nevertheless the experiments were performed blind to genotype.

For recordings from mice on an FVB and a hybrid genetic background, the experiments were performed in anaesthetized and awake animals, respectively. For recordings in anaesthetized experiments, the mice were sedated with an intraperioneal injection of chlorprothixene (4 mg/kg) and anaesthetized with urethane (1.2 g/kg). We monitored the toe-pinch reflex of the animal and supplemented additional urethane (0.2 – 0.3 g/kg) as needed. The animal was head-fixed in a stereotaxic apparatus and its body temperature was maintained at 37℃ with a heating blanket (FHC Inc.). A craniotomy (~ 1 mm diameter) was made above the monocular region of V1, and the dura was removed. Mice used in the awake experiments were first implanted with custom-made headplates at least 3 days before the recording. During the headplate implant, mice were anaesthetized with an intraperitoneal injection of the mixture of ketamine (50 mg/kg) and

meditomidine (0.6 mg/kg), and head-fixed in a stereotaxic apparatus. Body temperature was maintained at 37℃ with a heating blanket. A thin layer of tissue adhesive (Vetbond, 3M) was applied to the skull and a stainless-steel headplate was cemented to the skull with dental acrylic. A subcutaneous injection of carprofen (5 mg/kg) was injected after the surgery. For recordings in awake experiments, the headplate of the mouse was fixed to a holder attached to the stereotaxic apparatus and the mouse's body was restricted in a circular plastic tube. Following anaesthesia with isoflurane (~1%), a craniotomy (~ 1 mm diameter) was made above V1, and the exposed cortex was protected with a silicone elastomer (Kwik-Cast, World Precision Instruments). The mouse was allowed to recover from the anaesthesia for at least 2 hours, after which the animal was head-fixed to a holder via the headplate. The dura was removed before the recordings. After the experiments, the mouse was euthanized by an overdose of pentobarbital (0.5 g/kg) followed by cervical dislocation.

2. Electrophysiology.

The neural responses were amplified and filtered using a Cerebus 96-channel system (Blackrock microsystem). Local field potential and spiking signals were sampled at 2 kHz and 30 kHz, respectively. To detect the waveforms of spikes, we band-pass filtered the signals at 0.25 to 7.5 kHz and set a threshold at 4 s.d. of the background noise. Spikes were sorted offline with the Offline Sorter (Plexon Inc.) using cluster analysis of principal component amplitudes. Spike clusters were considered to be single units if few spikes (< 0.3%) had an interspike interval < 1 ms and $P < 0.05$ for multivariate analysis of variance tests on clusters[2]. To determine whether a single neuron was recorded by more than one

site in the electrode, we computed correlation coefficients (binned at 1 ms) between all pair-wise combinations of units in the same recording. Those pairs with a correlation coefficient > 0.1 were considered to contain duplicate units, and the unit with the lower firing rate in the pair was discarded[3].

3. Behaviour.

The 2AFC behavioural experiments were conducted in a custom-designed chamber (38 × 18 × 13 cm, L × W × H), with three ports positioned along the front wall facing an LCD monitor[4,5]. Nose-poke into each port was detected by the interruption of an infrared beam.

Mice were trained to perform the task using the following steps[5]. In step 1 (2 d), the center port of the chamber was blocked, and the mouse was rewarded with water (10 μl) by poking its nose into the two side ports in an alternating manner. A mouse received ~1.5 ml of water within each session. In step 2 (4 d), on alternate days, with the right (or left) port blocked by a barrier, the mouse poked its nose into the center port to trigger the presentation of a grating stimulus (a vertically oriented static grating, SF = 0.09 cycle/°, 100% contrast) on the left (or right) side of monitor, and learned to receive water (10 μl) from the left (or right) port. In step 3 (4 – 10 d), with all three ports open, the mouse initiated a trial by poking the center port, and a grating stimulus (SF = 0.09 cycle/°, 100% contrast) was presented on one side of the monitor. Choosing the port corresponding to the side the stimulus was presented was rewarded by water (4 – 5 μl), and choosing the incorrect port resulted in a timeout period (5 s). The stimulus disappeared after a maximum period of 20 s or after the mouse made the choice within this period. The mouse

advanced to the next step after reaching a correct rate of 80% for three consecutive sessions. In step 4 (3 – 8 d), the mouse was trained to perform SF detection task (SFs were randomly chosen from 0.05, 0.12, 0.24, 0.35, and 0.46 cycle/°, contrast = 100%) or contrast detection task (contrasts were randomly chosen from 20%, 30%, 40%, 65%, and 100%, SF = 0.09 cycle/°). In each session, we monitored the performance for each stimulus. Once the maximum performance in a session reached 75%, the training phase was over and the data in the following sessions were used for the analysis of visual detection. For SF detection task, a total of 5 sessions were measured for each mouse. For contrast detection task, a total of 4 sessions were measured for each mouse.

4. Analysis of neuronal responses.

We performed the following analyses to obtain orientation tuning and SF tuning curves for each unit[6]. First, for the responses to the stimulus set consisted of 12 directions and 9 SFs, we identified the orientation that produced the peak response by averaging across all SFs. Second, for the responses to different SFs at this orientation, we found the SF that produced peak response. Third, at this SF we constructed an orientation tuning curve, which was used for the analysis of orientation selectivity index (OSI) and preferred orientation for each unit. Finally, at the preferred orientation determined from the above orientation tuning curve, we constructed an SF tuning curve, which was used for the analysis of SF preference for each unit. For the responses to the stimulus set consisted of 12 directions and 7 TFs (or 12 directions and 8 contrasts), we performed analyses similar to those described above to construct a TF tuning curve (or a contrast response function), which was used for the analysis of preferred TF (or semi-saturation contrast) for each unit.

For the responses to bright or dark squares on a gray background, we computed the variance of the ON or OFF RF map at each time delay (bin size = 33.3 ms) after the stimulus onset. An SNR for the ON or OFF RF was computed as the ratio between the mean variance at delays 68 – 233 ms after stimulus onset and the mean variance at delays 467 – 600 ms[7]. A cell was included in the analysis of ON or OFF SNR if the SNR was > 1.5[8].

To determine the location of layer 4, we performed CSD analysis for the LFP responses to repeated full-screen flash for the 16 sites throughout cortical depth[2]. CSD was computed from the second spatial derivative of average LFP by the following equation[9,10]:

$$\frac{\partial^2 \phi}{\partial z^2} \approx \frac{\phi(z - n\Delta z) + \phi(z + n\Delta z) - 2\phi(z)}{(n\Delta z)^2} \qquad (1)$$

where ϕ is LFP, z is the coordinate of the recording sites, Δz is the distance between adjacent recording sites, and $n\Delta z$ is the differentiation grid (n = 2). Layer 4 was determined as the recording sites at the initial current sink. Layer 2/3 and layer 5/6 were those above and below layer 4, respectively. For the laminar analysis, we only used those recordings whose laminar locations could be determined from the CSD analysis.

For the response to drifting grating at the preferred SF, we computed the ratio between the amplitude of the first harmonic and the mean firing rate (F1/F0 ratio). Cells were classified as simple if F1/F0 $\geqslant$ 1 and complex if F1/F0 < 1[11].

Units were classified as broad-spiking or narrow-spiking based on the spike waveforms[6]. For each unit, all spikes were aligned by their troughs and averaged, and then the average waveform of each unit was interpolated[12]. Next the average waveforms

of all units were aligned by their troughs and normalized by trough depth. We computed two parameters: the width of trough to peak and the width of the peak at half-maximum of the peak amplitude[6,8,12]. Two clusters were identified using fuzzy c-means clustering.

References

1 Collins, A. L. *et al.* Mild overexpression of MeCP2 causes a progressive neurological disorder in mice. *Hum. Mol. Genet.* **13**, 2679-2689 (2004).

2 Zhu, Y., Qiao, W., Liu, K., Zhong, H. & Yao, H. Control of response reliability by parvalbumin-expressing interneurons in visual cortex. *Nat. Commun.* **6**, 6802 (2015).

3 Goard, M. & Dan, Y. Basal forebrain activation enhances cortical coding of natural scenes. *Nature Neurosci.* **12**, 1444-1449 (2009).

4 Busse, L. *et al.* The detection of visual contrast in the behaving mouse. *J. Neurosci.* **31**, 11351-11361 (2011).

5 Long, M., Jiang, W., Liu, D. & Yao, H. Contrast-dependent orientation discrimination in the mouse. *Sci. Rep.* **5**, 15830 (2015).

6 Niell, C. M. & Stryker, M. P. Highly selective receptive fields in mouse visual cortex. *J. Neurosci.* **28**, 7520-7536 (2008).

7 Yeh, C. I., Xing, D., Williams, P. E. & Shapley, R. M. Stimulus ensemble and cortical layer determine V1 spatial receptive fields. *Proc. Natl. Acad. Sci. USA.* **106**, 14652-14657 (2009).

8 Zhu, Y. & Yao, H. Modification of visual cortical receptive field induced by natural stimuli. *Cereb. Cortex* **23**, 1923-1932 (2013).

9 Mitzdorf, U. Current source-density method and application in cat cerebral cortex: investigation of evoked potentials and EEG phenomena. *Physiol. Rev.* **65**, 37-100 (1985).

10 Freeman, J. A. & Nicholson, C. Experimental optimization of current source-density technique for anuran cerebellum. *J. Neurophysiol.* **38**, 369-382 (1975).

11 Skottun, B. C. *et al.* Classifying simple and complex cells on the basis of response modulation. *Vision Res.* **31**, 1079-1086 (1991).

12 Mitchell, J. F., Sundberg, K. A. & Reynolds, J. H. Differential attention-dependent response modulation across cell classes in macaque visual area V4. *Neuron* **55**, 131-141 (2007).

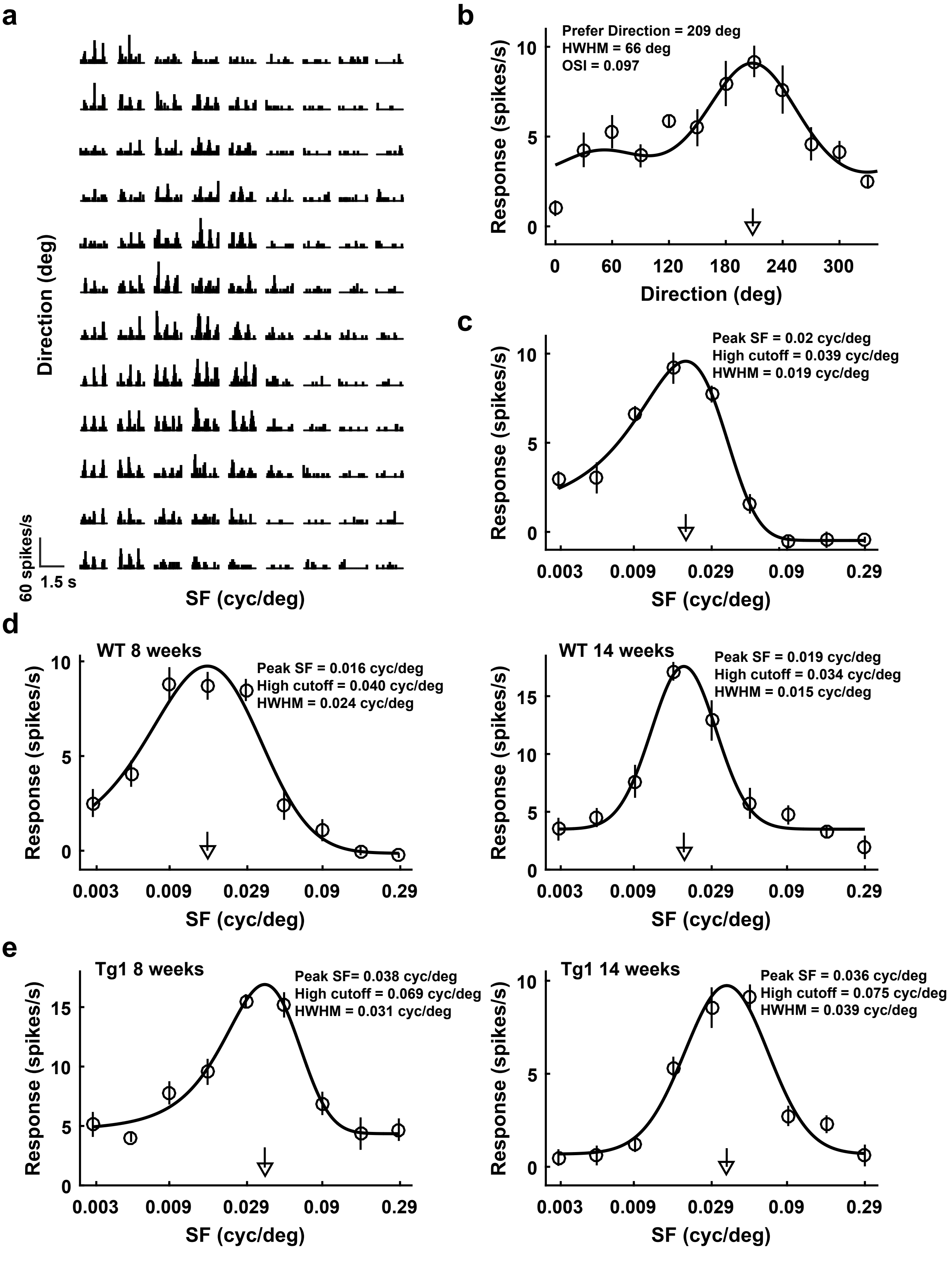

Figure S1. Responses of example V1 neurons to oriented gratings at different spatial frequencies. (a) Peri-stimulus time histograms (PSTHs) for an example V1 neuron from a WT mouse at 8 weeks. Bin size = 33.3 ms. Each small panel is the response to drifting grating at a specific direction and SF. Each row corresponds to a different direction (from upper to lower: 0° to 330°, spacing at 30°) and each column corresponds to a different SF (from left to right: 0.003, 0.005, 0.009, 0.016, 0.029, 0.05, 0.09, 0.16, and 0.29 cycle/°). (b) and (c), Orientation tuning and SF tuning extracted from the responses of the example neuron shown in (a). (d) SF tunings of two example V1 neurons from WT mice (left, 8 weeks; right, 14 weeks). (e) SF tunings of two example V1 neurons from Tg1 mice (left, 8 weeks; right, 14 weeks). Error bars, ± s.e.m. For panel b, arrow points to the preferred direction. For panel c, d, and e, arrow points to the peak SF.

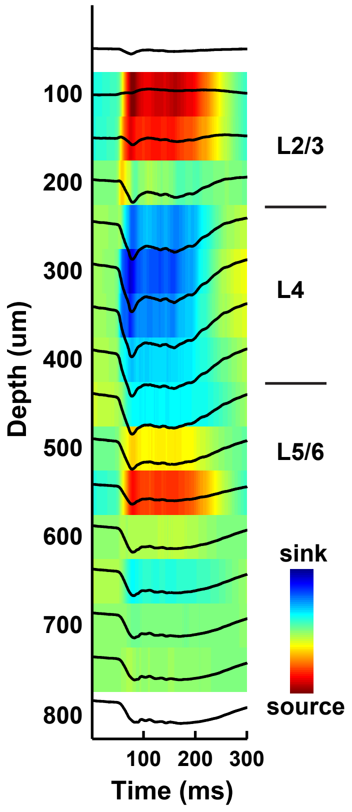

Figure S2. An example of CSD analysis to identify cortical layers. Black traces, flash-evoked LFP responses at different recording sites along the cortical depth. Layer 4 was determined as the recording sites at the initial current sink. Layer 2/3 and layer 5/6 were those above and below layer 4, respectively.

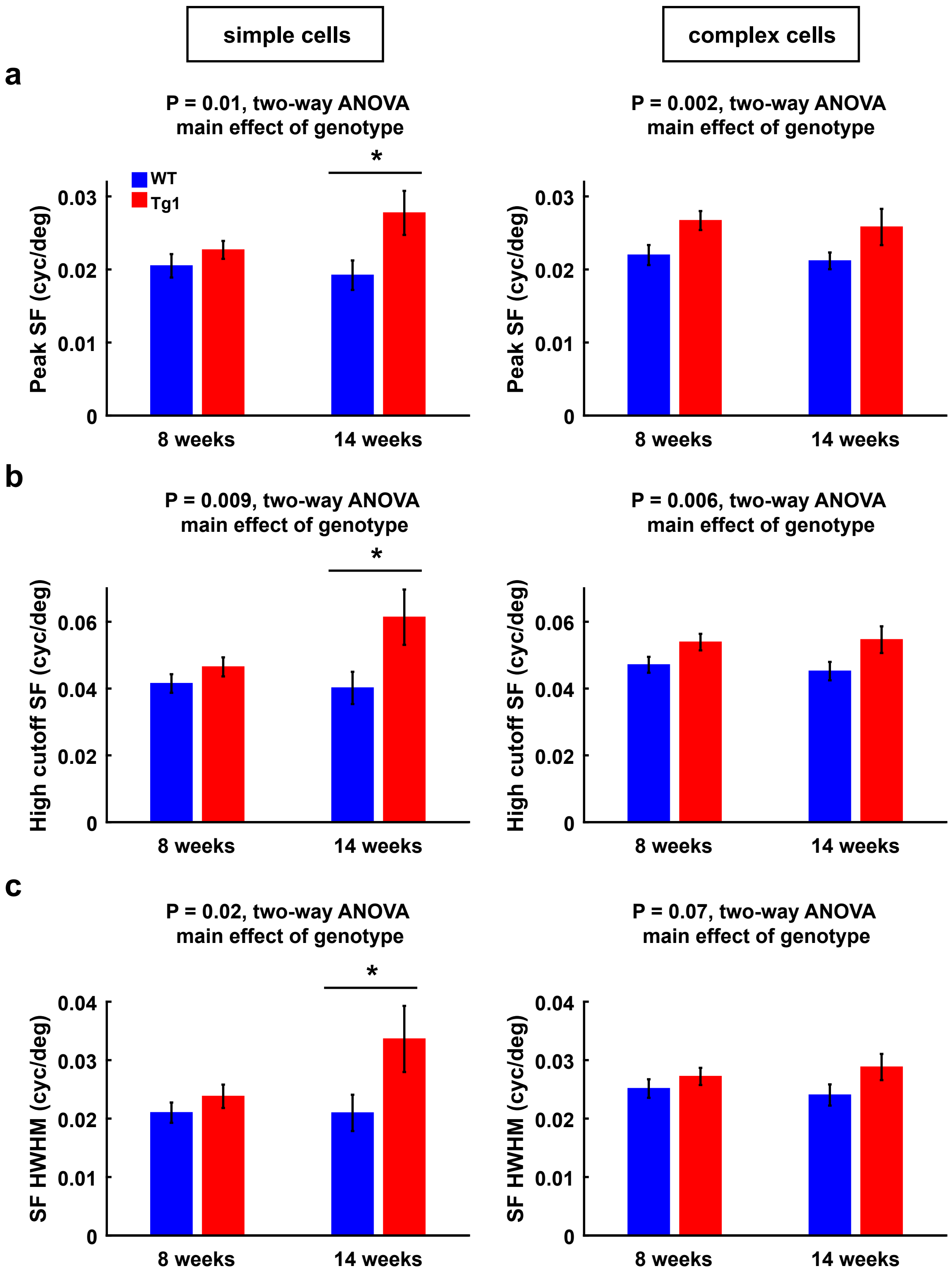

Figure S3. Comparison of spatial frequency preference between V1 neurons in WT and Tg1 mice, for simple and complex cells, respectively. (a) Comparison of mean preferred SFs between WT and Tg1 mice for simple cells (left) and complex cells (right). P = 0.01 and 0.002 for simple cells and complex cells, respectively, two-way ANOVA, main effect of genotype. (b) Comparison of mean high cutoff SFs between WT and Tg1 mice for simple cells (left) and complex cells (right). P = 0.009 and 0.006 for simple cells and complex cells, respectively, two-way ANOVA, main effect of genotype. (c) Comparison of mean HWHMs between WT and Tg1 mice for simple cells (left) and complex cells (right). P = 0.02 and 0.07 for simple cells and complex cells, respectively, two-way ANOVA, main effect of genotype. For comparison between WT and Tg1 mice at each age, statistical significance was determined from two-way ANOVA followed by Tukey's multiple comparison test. * $P < 0.05$, Error bars, ± s.e.m. For simple cells, n = 85 and 76 for WT mice at 8 and 14 weeks, respectively, n = 76 and 54 for Tg1 mice at 8 and 14 weeks, respectively. For complex cells, n = 170 and 191 for WT mice at 8 and 14 weeks, respectively, n = 203 and 99 for Tg1 mice at 8 and 14 weeks, respectively.

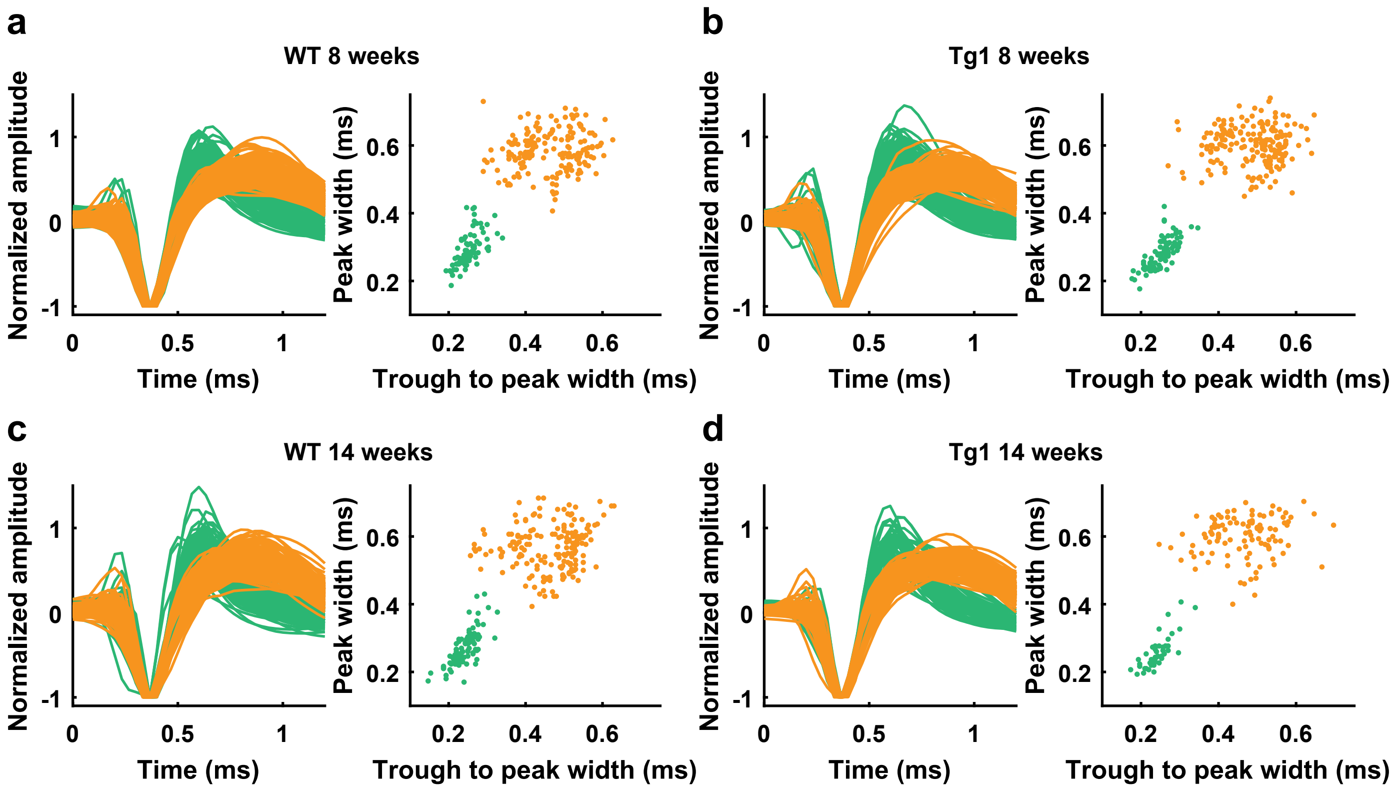

Figure S4. Spike waveform classification for V1 neurons in WT and Tg1 mice. (a) Spike waveforms for all units from WT mice at 8 weeks. Left, average spike waveforms for all units in response to oriented gratings at different SFs. Right, scatter plot of spike waveform parameters for all units. Green, narrow-spiking; orange, broad-spiking. (b) – (c) Spike waveforms for all units from Tg1 mice at 8 weeks, WT mice at 14 weeks, and Tg1 mice at 14 weeks, respectively. Same as described in (a).

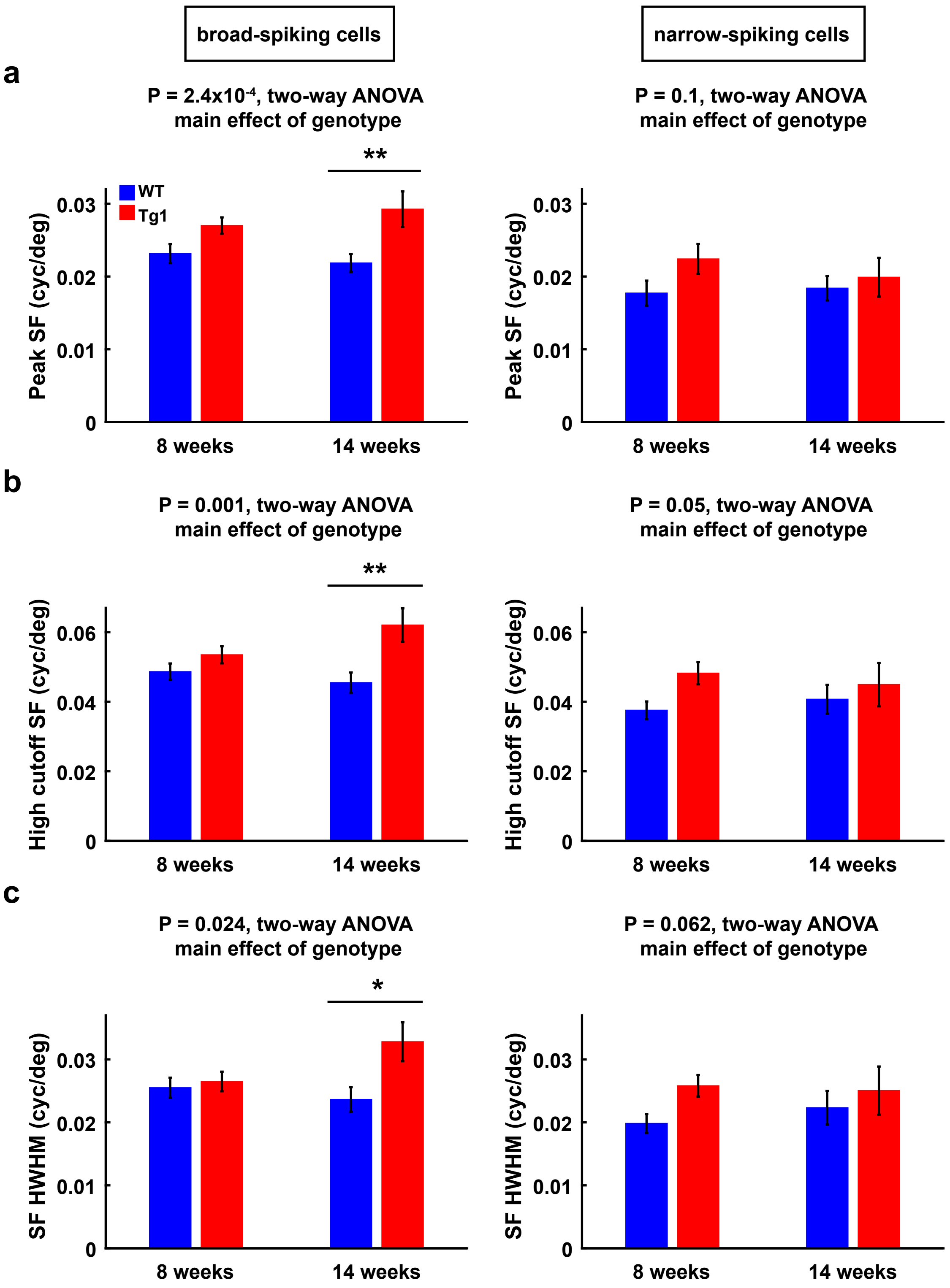

Figure S5. Comparison of spatial frequency preference between V1 neurons in WT and Tg1 mice, for broad-spiking and narrow-spiking cells, respectively. (a) Comparison of mean preferred SFs between WT and Tg1 mice for broad-spiking cells (left) and narrow-spiking cells (right). $P = 2.4 \times 10^{-4}$ and 0.1 for broad-spiking cells and narrow-spiking cells, respectively, two-way ANOVA, main effect of genotype. (b) Comparison of mean high cutoff SFs between WT and Tg1 mice for broad-spiking cells (left) and narrow-spiking cells (right). P = 0.001 and 0.05 for broad-spiking cells and narrow-spiking cells, respectively, two-way ANOVA, main effect of genotype. (c) Comparison of mean HWHMs between WT and Tg1 mice for broad-spiking cells (left) and narrow-spiking cells (right). P = 0.024 and 0.062 for broad-spiking cells and narrow-spiking cells, respectively, two-way ANOVA, main effect of genotype. For comparison between WT and Tg1 mice at each age, statistical significance was determined from two-way ANOVA followed by Tukey's multiple comparison test. * $P < 0.05$, ** $P < 0.01$, Error bars, ± s.e.m. For broad-spiking cells, n = 177 and 172 for WT mice at 8 and 14 weeks, respectively, n = 194 and 108 for Tg1 mice at 8 and 14 weeks, respectively. For narrow-spiking cells, n = 78 and 95 for WT mice at 8 and 14 weeks, respectively, n = 85 and 45 for Tg1 mice at 8 and 14 weeks, respectively.

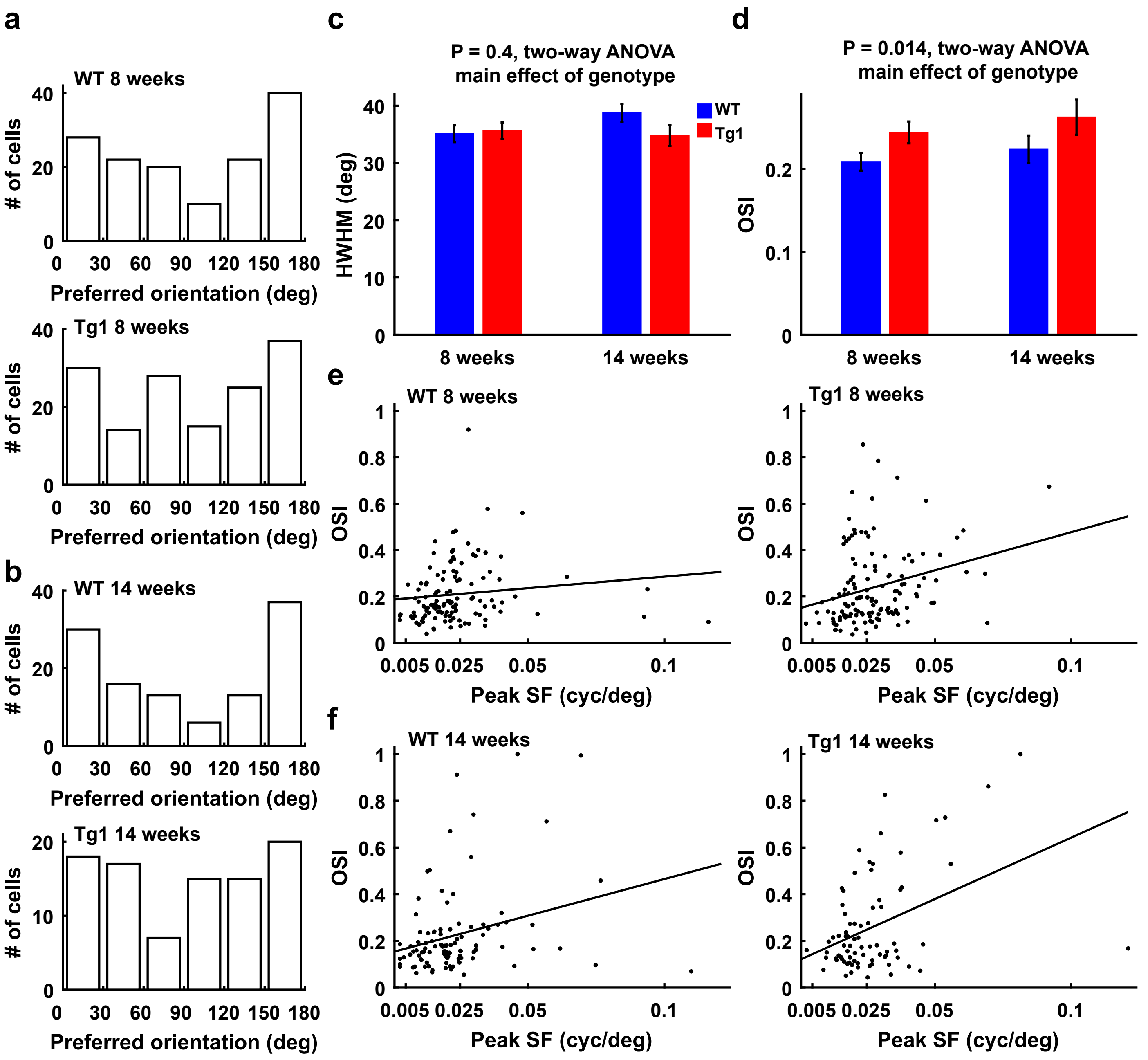

Figure S6. Comparison of orientation tuning properties between V1 neurons in WT and Tg1 mice. (a) The distributions of preferred orientations for V1 neurons in 8-week WT and Tg1 mice were not significantly different (P = 0.5, n = 142 from 26 WT mice, n = 149 from 25 Tg1 mice, X^2 test). (b) The distributions of preferred orientations for V1 neurons in 14-week WT and Tg1 mice were not significantly different (P = 0.96, n = 115 from 22 WT mice, n = 92 from 12 Tg1 mice, X^2 test). (c) HWHMs of orientation tuning were not significantly different between V1 neurons in WT and Tg1 mice. P = 0.4, two-way ANOVA, main effect of genotype. (d) OSIs of V1 neurons in Tg1 mice were significantly higher than those in WT mice. P = 0.014, two-way ANOVA, main effect of genotype. (e) Left panel, OSI against preferred SF for V1 neurons from WT mice at 8 weeks (r = 0.12, P = 0.17, n = 123 from 25 mice). Right panel, OSI against preferred SF for V1 neurons from Tg1 mice at 8 weeks (r = 0.27, P = 0.002, n = 132 from 25 mice). (f) OSI against preferred SF for V1 neurons from WT mice at 14 weeks (r = 0.29, P = 0.004, n = 100 from 22 mice). Right panel, OSI against preferred SF for V1 neurons from Tg1 mice at 14 weeks (r = 0.42, P = $9.8X10^{-5}$, n = 79 from 12 mice). The line indicates the linear regression between OSI and preferred SF. Error bars, ± s.e.m.

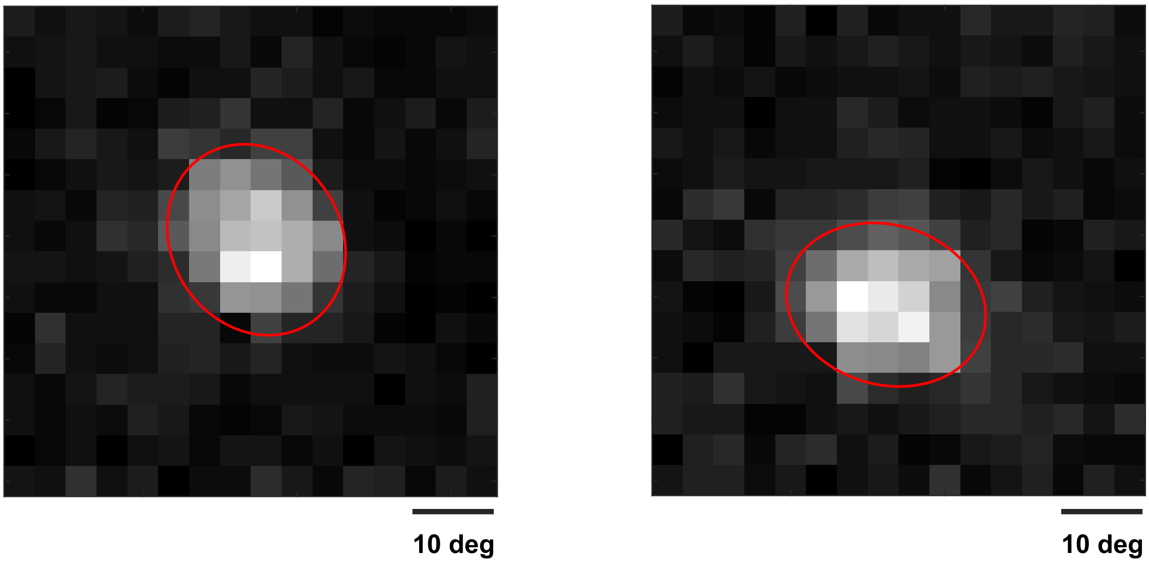

Figure S7. Examples of receptive field maps. Left, spatial RF map for an example V1 neuron from a WT mouse at 8 weeks. Right, spatial RF map for an example V1 neuron from a Tg1 mouse at 8 weeks. Ellipse, contour of Gaussian fit at 2 s.d.

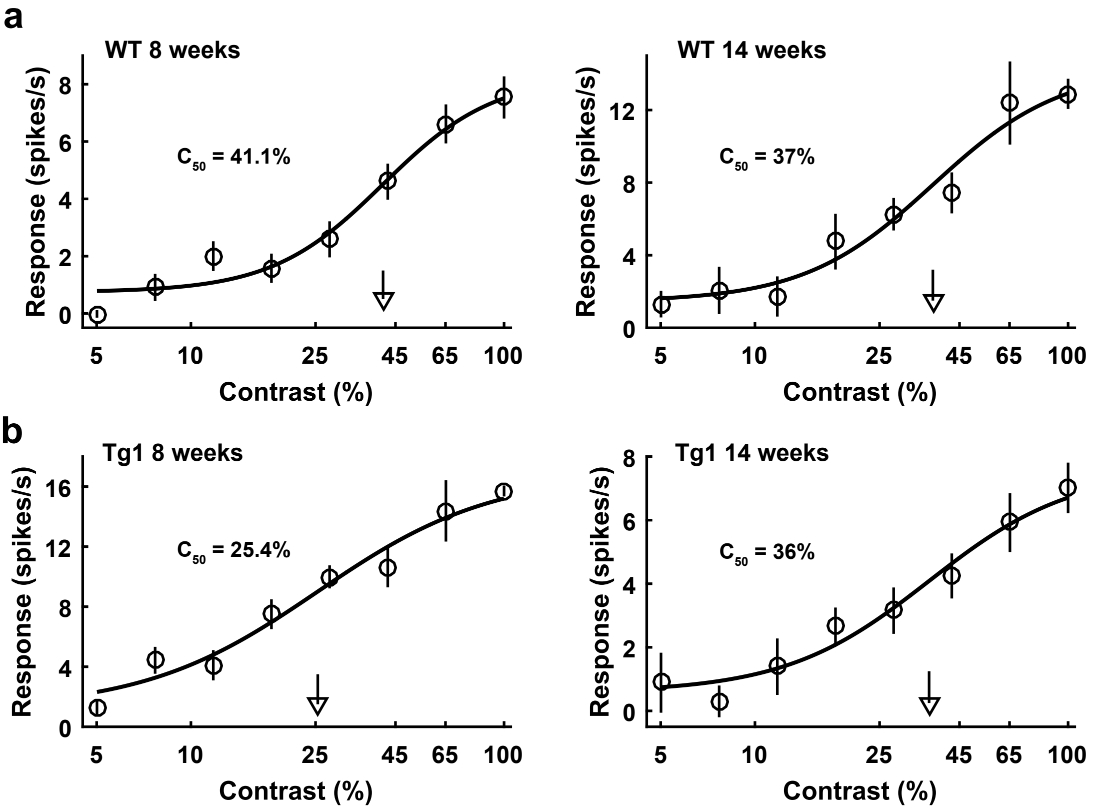

Figure S8. Examples of contrast response functions. (a) Contrast response functions of two example V1 neurons from WT mice (left, 8 weeks; right, 14 weeks). (b) Contrast response functions of two example V1 neurons from Tg1 mice (left, 8 weeks; right, 14 weeks). Arrow points to the C_{50}.

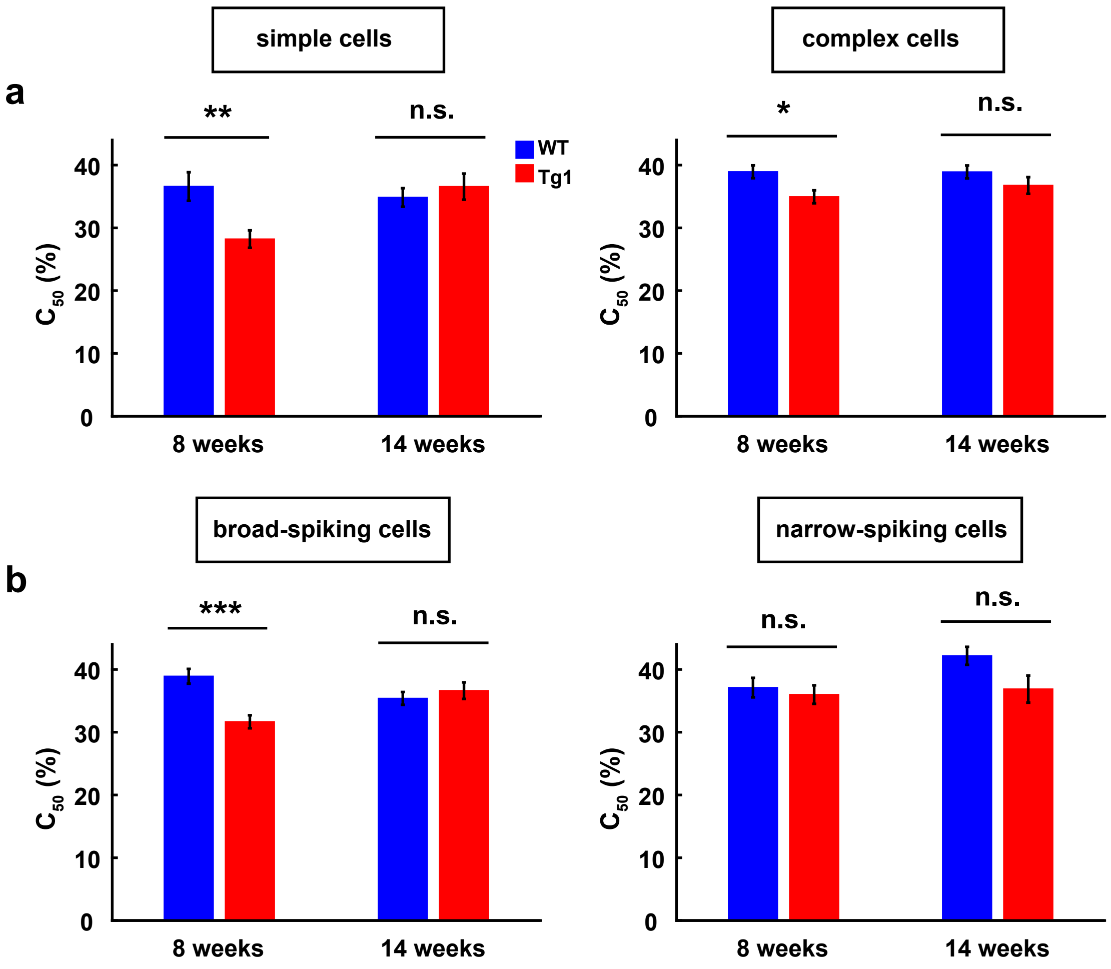

Figure S9. Comparison of contrast sensitivity between V1 neurons in WT and Tg1 mice for different types of cells. (a) Comparison of C_{50} between WT and Tg1 mice for simple cells (left) and complex cells (right). (b) Comparison of C_{50} between WT and Tg1 mice for broad-spiking cells (left) and narrow-spiking cells (right). Error bars, ± s.e.m., * $P < 0.05$, ** $P < 0.01$, *** $P < 0.001$, two-way ANOVA followed by Tukey's multiple comparison test. For simple cells, n = 44 and 80 for WT mice at 8 and 14 weeks, respectively, n = 71 and 60 for Tg1 mice at 8 and 14 weeks, respectively. For complex cells, n = 143 and 178 for WT mice at 8 and 14 weeks, respectively, n = 188 and 113 for Tg1 mice at 8 and 14 weeks, respectively. For broad-spiking cells, n = 132 and 172 for WT mice at 8 and 14 weeks, respectively, n = 173 and 127 for Tg1 mice at 8 and 14 weeks, respectively. For narrow-spiking cells, n = 55 and 86 for WT mice at 8 and 14 weeks, respectively, n = 86 and 46 for Tg1 mice at 8 and 14 weeks, respectively.

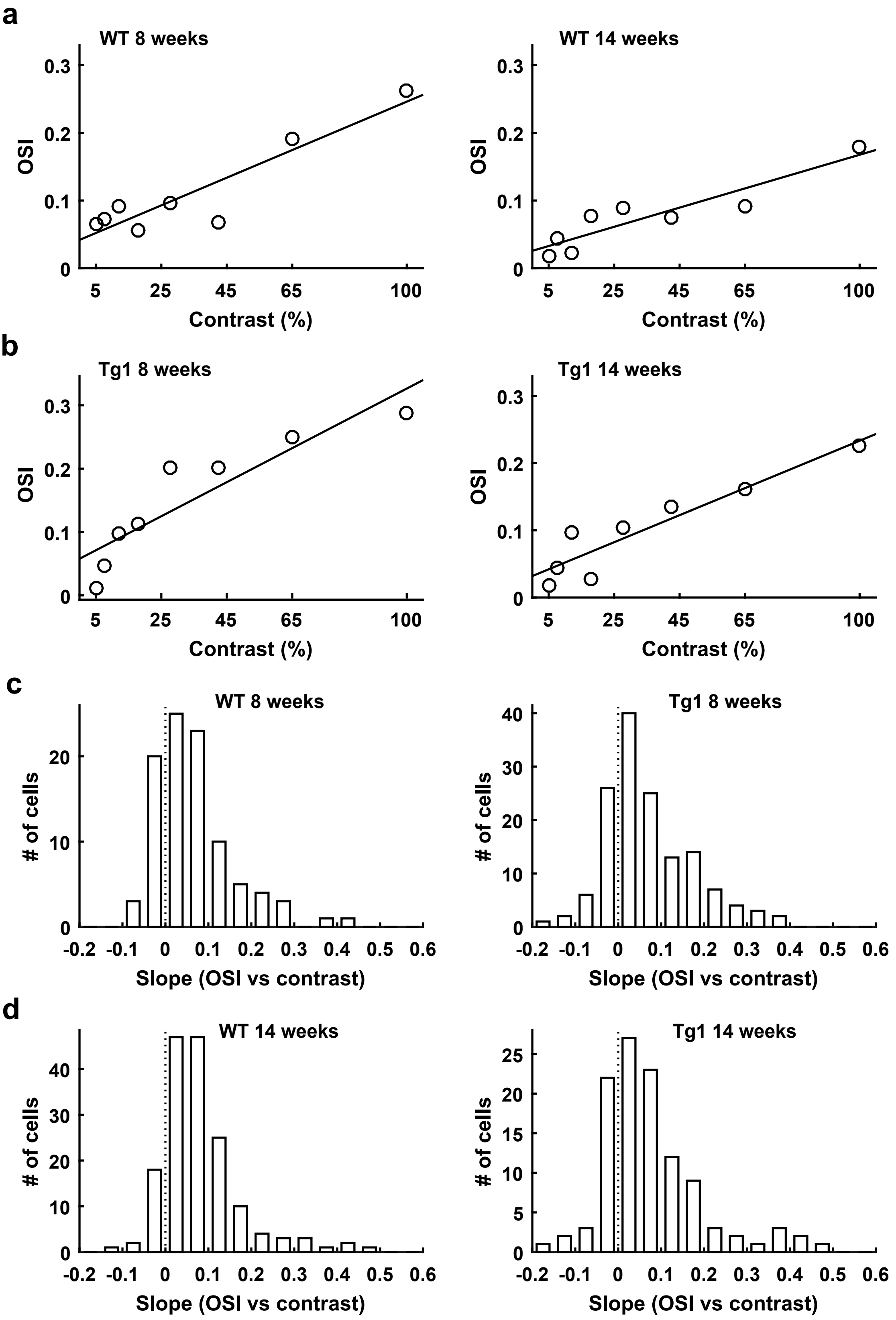

Figure S10. Relationship between OSI and preferred SF for V1 neurons in WT and Tg1 mice. (a) OSI against contrast for example V1 neurons of a 8-week WT mouse (left) and a 14-week WT mouse (right). (b) OSI against contrast for example V1 neurons of a 8-week Tg1 mouse (left) and a 14-week Tg1 mouse (right). The line indicates the linear regression between OSI and contrast. (c) Histograms showing the distributions of the slopes of linear regression between OSI and contrast for V1 neurons in WT (left, n = 95 neurons from 18 animals) and Tg1 mice (right, n = 143 neurons from 22 animals) at 8 weeks. (d) Histograms showing the distributions of the slopes for V1 neurons in WT (left, n = 164 neurons from 26 animals) and Tg1 mice (right, n = 111 neurons from 17 animals) at 14 weeks.
